# Deciphering the mechanisms of Yinlan Tiaozhi capsule in treating hyperlipidemia by combining network pharmacology, molecular docking and experimental verification

**DOI:** 10.1038/s41598-023-33673-3

**Published:** 2023-04-19

**Authors:** Guanlin Xiao, Zixuan Hu, Canchao Jia, Minjuan Yang, Dongmei Li, Aili Xu, Jieyi Jiang, Zhao Chen, Yangxue Li, Sumei Li, Weitao Chen, Jingnian Zhang, Xiaoli Bi

**Affiliations:** 1 Guangdong Province Engineering and Technology Research Institute of Traditional Chinese Medicine/Guangdong Provincial Key Laboratory of Research and Development in Traditional Chinese Medicine, Guangzhou, People’s Republic of China; 2 School of the Fifth Clinical Medicine, Guangzhou University of Chinese Medicine, Guangzhou, 510405 People’s Republic of China

**Keywords:** Computational biology and bioinformatics, Drug discovery, Diseases, Chemistry

## Abstract

Yinlan Tiaozhi capsule (YLTZC) has been widely used to treat hyperlipidemia (HLP). However, its material basis and underlying pharmacological effects remain unclean. The current study aimed to explore the mechanisms involved in the treatment of YLTZC on HLP based on network pharmacology, molecular docking, and experimental verification. Firstly, UPLC-Q-TOF–MS/MS was used to comprehensively analyze and identify the chemical constituents in YLTZC. A total of 66 compounds, mainly including flavonoids, saponins, coumarins, lactones, organic acids, and limonin were characterized and classified. Simultaneously, the mass fragmentation pattern of different types of representative compounds was further explored. By network pharmacology analysis, naringenin and ferulic acid may be the core constituents. The 52 potential targets of YLTZC, including ALB, IL-6, TNF, and VEGFA, were considered potential therapeutic targets. Molecular docking results showed that the core active constituents of YLTZC (naringenin and ferulic acid) have a strong affinity with the core targets of HLP. Lastly, animal experiments confirmed that naringenin and ferulic acid significantly upregulated the mRNA expression of ALB and downregulated the mRNA expression of IL-6, TNF, and VEGFA. In sum, the constituents of YLTZC, such as naringenin and ferulic acid, might treat HLP by regulating the mechanism of angiogenesis and inhibiting inflammatory responses. Furthermore, our data fills the gap in the material basis of YLTZC.

## Introduction

Hyperlipidemia (HLP) is one of the most common disorders of lipid metabolism, which is closely related to metabolic syndrome, diabetes, obesity, and arteriosclerosis, and is a major risk factor for cardiovascular and cerebrovascular diseases^1,2^. The main drugs currently used in the treatment of HLP include statins, fibrates, and cholesterol absorption inhibitors, which have caused many side effects for patients during treatment, such as gastrointestinal tract issues, myopathy, and rhabdomyolysis^3^. Therefore, it is necessary to find alternative medicines for the treatment of HLP.

Yinlan Tiaozhi capsule (YLTZC) is a classic traditional Chinese medicine (TCM) prescription and patent formula. It has been clinically used for treating HLP and consists of *Citri Grandis Exocarpium*, *Ginkgo Folium*, *Gynostemma pentaphyllum*, and propolis^4–7^. The clinical trials of new drug approval (2012L01011) have been successfully admitted by National Medical Products Administration (NMPA), and is currently in phase II clinical stage. Data from previous studies revealed that the YLTZC showed obvious anti-HLP activity and could regulate lipid levels in various animal models of experimental HLP, including mice, rats, and New Zealand rabbits. The experimental results showed that YLTZC remarkably lowered the levels of total cholesterol (TC), triglycerides (TG), and low-density lipoprotein cholesterol (LDL-c) in HLP rabbits, mice, and rats, while increasing the levels of high-density lipoprotein cholesterol (HDL-c), and the anti-HLP effect of YLTZC might be related to inhibiting PXR expression, promoting bile acid excretion and RCT processes, and enhancing TG hydrolysis^4–7^. In addition, in the treatment of type 2 diabetes mellitus, YLTZC was performed to prohibit the FA β-oxidation, synthesis of cholesterol and phospholipids, gluconeogenesis, and inflammation level, as well as promote TG hydrolysis, glycolysis, and blood circulation^8^. However, there are still some problems in the related research of YLTZC. On the one hand, its pharmacodynamic substance basis and quality control system are still unclear. On the other hand, most of the current reports are limited to a single constituent and single target, while for the treatment of HLP, YLTZC plays a role primarily by acting on multiple ingredients, targets, and pathways, which means that its potential pharmacological effects are complex. The above-mentioned problems limited the systematic understanding of the mechanism of action of YLTZC. Therefore, it is necessary to establish a rapid and effective method to investigate the material basis in YLTZC, as well as the relationship and mechanism of action between the core active constituents and the core targets.

Ultra-performance liquid chromatography-quadrupole time-of-flight mass spectrometry (UPLC-Q-TOF–MS/MS), with high resolution, high sensitivity, and short analysis time, has become a powerful tool for the qualitative analysis in complex systems of TCM^9^. With the rapid and continuous development of bioinformatics, network pharmacology has become an important approach in the study of TCM, which provides a basis for systematically understanding the complexity between drug action and disease and elucidating potential therapeutic mechanisms^10,11^.

**Figure 1:**
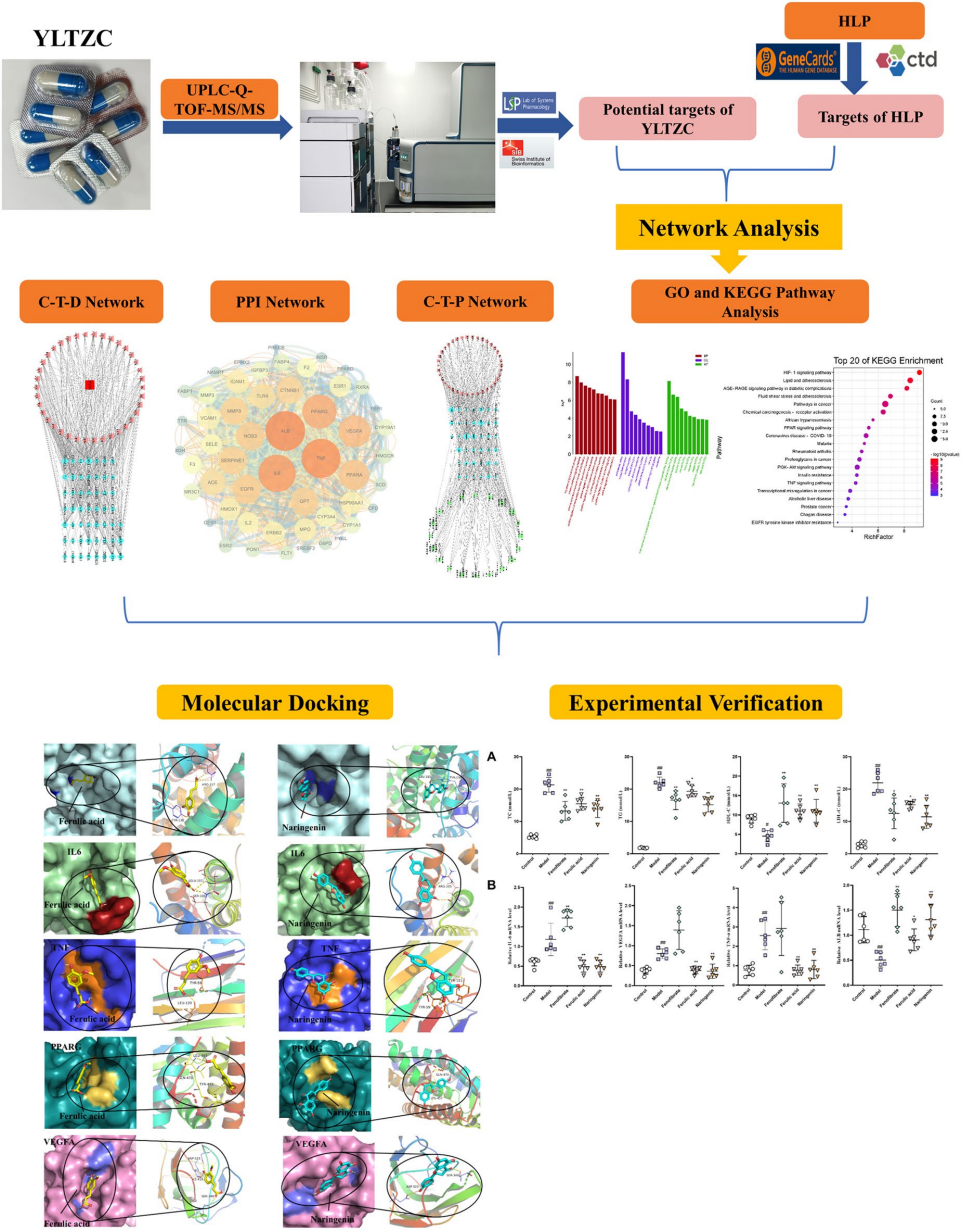
Workfow diagram of the network pharmacology-based analysis of YLTZC in the treatment of HLP.

In this research, we revealed the effective ingredients and predicted the potential targets and signaling pathways of YLTZC for the treatment of HLP by integrating the chemical profile, network pharmacology, molecular docking, and experiment verification. The detailed flowchart is shown in Fig. 1. This study provides a theoretical basis for investigating the pharmacodynamic material basis and quality control of YLTZC in treating HLP, which will contribute to its development and application.

## Materials and methods

### Chemicals, reagents and materials

Methanol, formic acid, and acetonitrile of MS grade were purchased from Thermo Fisher Scientific (United States), and laboratory-deionized water generated by a Milli-Q system (United States) was used in all experiments.

YLTZC (Batch No. 20211101) was provided by Guangdong Efong Pharmaceutical Co. Ltd. (Foshan, China). Reference substances including vicenin-2, luteolin, naringenin, isomeranzin, ferulic acid and pinocembrin were all purchased from Chengdu Herbpurify Co., Ltd (Chengdu, China). Gallic acid, protocatechuic acid, rutin, naringin, ginkgolide A, ginkgolide B, limonin, and chrysin were all purchased from National Institutes for Food and Drug Control (Beijing, China). Triton WR-1339 was purchased from Sigma-Aldrich (Lot#MKCC6730; Shanghai, China).

### Chemical profile

#### Standards and sample preparation

YLTZC was ground into powder, 0.25 g of powder was accurately weighed and extracted with 50 ml 50% aqueous ethanol under ultrasonication for 30 min. The extract solution was centrifuged at 12,000 rpm for 10 min at 25 °C. And then, the supernatant was filtrated through 0.22 μm filter membrane and 1.0 μl of filtrate was injected into UPLC-Q-TOF–MS/MS of analysis. 14 reference standards (gallic acid, protocatechuic acid, vicenin-2, rutin, ferulic acid, naringin, ginkgolide A, ginkgolide B, luteolin, naringenin, isomeranzin, limonin, chrysin, and pinocembrin) were weighed in appropriate amounts, dissolved in methanol and filtered by 0.22 μm filter membrane to prepare 0.3 mg/ml stock solution, respectively.

#### Liquid chromatography–mass spectrometry analysis

Liquid chromatography analysis was conducted by using a SHIMADZU ExionLC system (Japan). Chromatographic separation was used Waters ACQUITY BEH C_18_ column (100 mm × 2.1 mm, 1.7 μm) at 30 °C, with mobile phases A (0.1% formic acid) and B (acetonitrile). The flow rate was 0.3 ml/min, and the gradient profile was showed as follows: 0–3 min, 8–25% B; 3–12 min, 25–47% B; 12–25 min, 47–79% B; 25–32 min, 79–95% B; 32–35 min, 95% B; 35–36 min, 95–8% B. The sample injection volume was 1 μl.

The MS analysis was acquired using an AB SCIEX X500R Q-TOF–MS/MS system (United States) with an electrospray ionization (ESI). The results of the mass spectrometry optimization conditions are as follows: ion voltage: − 4.0 kV and + 5.5 kV, Gas1 (nebulizer gas): 55 psi; Gas2 (heater gas): 55psi; curtain gas: 35 psi; declustering potential voltage: 80 V; ion source temperature: 500 °C; collision energy: 60 V; collision energy spread: 15 V; scan range: *m*/*z* 50–1000. Data were collected in information-dependent acquisition mode, and the instrument was recalibrated every four hours in order to exclude dynamic background. All data collected and analyzed by SCIEX OS v2.1 software (Framingham, MA, United States, 2021).

#### Establishment of a chemical constituents library for YLTZC

The chemical constituents of the 4 herbs in YLTZC were collected from existing databases, including CNKI (https://www.cnki.net/), SciFinder (https://scifinder.cas.org/) and literature searches, etc. Then, a self-built database of YLTZC chemical constituents containing component names and molecular formula information was established (Supplementary Table S1). MS data was imported into SCIEX OS v2.1 for analysis. Chemical identifications were combined with reference to relevant literatures and standards, precise relative molecular masses, mass spectrometry fragment information, and mass spectrometry library (Natural Products HR-MS/MS Spectral Library, Version 1.0, AB Sciex, United States).

### Network pharmacology analysis

#### Active constituent screening of YLTZC

The SwissADME tool (http://www.swissadme.ch/) was used for analyzing active constituents with absorption, distribution, metabolism, and excretion (ADME) properties and druglikeness evaluation to screen for active constituents with potential therapeutic effects. We applied in this study screening criteria of (1) pharmacokinetics “high” and (2) druglikeness (DL) with more than two “yes”^12^.

#### Target network analysis

In this research, the targets of active constituents in YLTZC were searched by Swiss Target Prediction database (http://www.swisstargetprediction.ch/), and targets with probability greater than 0 were selected. The HLP-related targets were selected from CTD (http://ctdbase.org/) and GeneCards (https://www.genecards.org/) database using the keywords “hyperlipidemias” and “hyperlipidemia”. Venny 2.1.0 (https://bioinfogp.cnb.csic.es/tools/venny/) was used to identify the overlapped targets between YLTZC-related targets and HLP-related targets. The common targets were entered into the STRING database (https://string-db.org), and biological species was set to Homo sapiens and the confidence > 0.4^13^. Cytoscape 3.7.1 was used to construct the component-target-disease (C-T-D) network and Protein–Protein Interaction (PPI) Network^14^.

#### Enrichment analysis and component-target-pathway network

The GO and KEGG pathway enrichment analysis of the common targets involved in the PPI network were performed using the DAVID 6.8 database (https://david.ncifcrf.gov/), and were selected for “OFFICE_GENE_SYMBOL” and “Homo sapiens”^15–17^. The GO enrichment analysis included the following 3 categories: biological process (BP), cellular component (CC), and molecular function (MF). The top 10 enriched GO entries and the top 20 enriched KEGG pathways were selected, and uploaded to the Bioinformatics (http://www.bioinformatics.com.cn/) cloud platform for data visualization. Cytoscape 3.7.1 was used to construct the component-target-pathway (C-T-P) network, and the core active constituents were obtained through the C-T-P network.

### Molecular docking validation

#### Molecular docking of active constituent-core target

The core targets and the core active constituents were obtained from the PPI and C-T-P network for molecular Docking. The protein PDB files of the core targets were obtained from the RSCB PDB online platform (https://www.rcsb.org/) and the 3D structure of compounds were acquired from the PubChem database (https://pubchem.ncbi.nlm.nih.gov/)^18^, and docking validation after hydrogenation and dehydration was carried out by AutoDockTools (version 1.5.6) and AutoDock Vina (version 1.1.2). Proteins should be selected from human proteins with one or more cocrystallized ligands and crystal structures with small “resolution” value^19^. Due to the still unclean functional binding sites of HLP-related targets to ingredients of YLTZC, all binding sites were restricted to the docking pockets region predicted by DeepSite software (https://www.playmolecule.com)^20^. Lastly, the molecular docking results were visualized into Pymol (2.5.0) software.

### Experiments evaluation

#### Animal models and ethics statements

All experiment procedures were compiled with the NIH recommendations for the use and care of animals. The animal experimental protocols were conducted in conformity with the policies and procedures of the animal ethics committee of Guangdong Provincial Engineering Technology Institute of Traditional Chinese Medicine (Guangzhou, China), and all animal experiments were performed in accordance with relevant ARRIVE guidelines. The 30 male KM mice weighing between 18 and 22 g were obtained from the Guangdong Medical Laboratory Animal Center (Guangzhou, China). All animals were housed in barrier system at standard room temperature and a 12 h light/dark cycle conditions and fed normal food and water. The food was supported by Guangdong Medical Laboratory Animal Center (Guangzhou, China). The experiment mice were divided into the following five groups with six mice in each: control group, model group, fenofibrate group (26 mg/kg), ferulic acid group (50 mg/kg), and naringenin group (50 mg/kg). The administration groups were given corresponding drugs by gavage, once a day, for 5 days. On the third day of administration, triton WR-1339 (480 mg/kg) was administered intramuscularly to all groups except the normal control group to establish a model of acute HLP. On the fifth day, one hour after gavage administration, all mice were anesthetized with isoflurane, sacrificed by inner canthus artery exsanguination, and the organs were retained for analysis.

#### Biochemical parameters

The serum sample was obtained by centrifugation of blood at 3000 rpm at 4 °C for 10 min and preserved at − 80 °C until analysis. TC, TG, HDL-c and LDL-c levels of serum were measured by Microplate Reader (Varioskan Flash, Thermo, United States) with assay kits were purchased from Nanjing Jiancheng Bioengineering Institute (Nanjing, China).

#### Quantitative real-time PCR

About 50 mg of liver was weighed and transferred into a 1.5 ml grinding tube. After adding 500 µl Trizol reagent (Beijing, China) and 2 grinding beads to the grinding tube, the liver was crashed by a freezing grinder (Shanghai Jingxin, China). The grinding liquid was centrifuged (4 °C, 10 min, 12,000 rpm) and the supernatant (about 400 µl) was transferred into a 1.5 mL centrifuge tube. 100 µl chloroform was added to the centrifuge tube and they were fully mixed by sharking. After centrifuging at 12,000 rpm at 4 °C for 10 min, the supernatant (about 200 µl) and the equivalent isopropyl alcohol were added into a 1.5 mL centrifuge tube and stored overnight in a − 20 °C refrigerator. The crushed total RNA was settled at the bottom of the centrifuge tube after centrifugation at 12,000 rpm at 4 °C for 10 min. After washing with 75% ethanol 2 times, the pure total RNA was gained and dissolved in 50 µl RNase-free water. The content of total RNA was measured by UV and 500 ng total RNA was reverse transcribed by using the Evo M-MLV RT Premix for qPCR (Accurate Biology, China). RT-qPCR reactions were performed on iQ5 Multicolor Real-Time PCR detection system (BIO-RAD, Hercules, California, United States) with SYBR Green Dye detection. The relative gene expression was determined by the 2^−ΔΔCt^ method, and Gene-expression data were normalized to that of the internal control GAPDH. The primer sequences are shown in Table 1.

**Table 1:** Primer sequence formation.

Gene name	Forward primer (5′–3′)	Reverse primer (5′–3′)
IL-6	AGTTGTGCAATGGCAATTCTGA	CTCTGAAGGACTCTGGCTTTGTC
TNF	CCCTCACACTCACAAACCACC	CTTTGAGATCCATGCCGTTG
ALB	AACAAGAGCCCGAAAGAAACG	CTGGCAACTTCATGCAAATAGTG
VEGFA	GTAACGATGAAGCCCTGGAGTG	TCACAGTGAACGCTCCAGGAT
GAPDH	CCTCGTCCCGTAGACAAAATG	TGAGGTCAATGAAGGGGTCGT

### Statistical analysis

Results were expressed as the mean ± standard deviation (SD), and analyzed by SPSS 20.0 software. Statistical significance was assessed using one-way analysis of variance (ANOVA). The value of *p* < 0.05 was considered statistically significant.

## Results

### Identification and characterization of chemical constituent

The mass spectrum data of YLTZC were gained by UPLC-Q-TOF–MS/MS. As shown in Table 2, a total of 66 chemical constituents were characterized by reference standards, reference literature, mass fragments, and mass spectrometry databases^21–32^. The main chemical constituent cluster was composed of 42 flavonoids, 6 saponins, 4 coumarins, 4 lactones, 9 organic acids and 1 limonin. Meanwhile, the identified constituents were attributed and classified based on the sources of their medicinal materials. Among them, 14 constituents (peak 1, 2, 5, 9, 13, 19, 22, 23, 24, 28, 38, 39, 42, and 44) were determined by comparing with the reference standards. An additional 52 constituents were identified by comparing relevant literatures and mass fragmentation patterns.

**Table 2:** Identification of chemical constituents of YLTZC by UPLC-Q–TOF–MS/MS.

Name	*t*_R_ (min)	Compound	Formula	*m*/*z*	*Δ* (ppm)	MS/MS fragments	Origin	Refs.
N1	1.85	Gallic acid*	C_7_H_6_O_5_	169.014 1 [M − H]^−^	− 0.8	125.024 6 [M − H-CO_2_]^−^	B/D	^23,24^
N2	2.97	Protocatechuic acid*	C_7_H_6_O_4_	153.018 9 [M − H]^−^	− 2.7	109.028 5 [M − H-CO_2_]^−^, 91.018 9 [M − H-CO_2_-H_2_O]^−^	A/D	^23,24^
N3	4.54	4-Hydroxycinnamic acid	C_9_H_8_O_3_	163.040 1 [M − H]^−^	0	119.050 1 [M − H-CO_2_]^−^	B/D	^24^
N4	4.82	Vanillic acid	C_8_H_8_O_4_	169.049 9 [M + H]^+^	2.2	151.041 4 [M + H-H_2_O]^+^, 123.045 2 [M + H-H_2_O-CO]^+^	B	^32^
N5	5.28	Vicenin-2*	C_27_H_30_O_15_	593.148 9 [M − H]^−^	− 3.9	473.105 3 [M − H-C_4_H_8_O_4_]^−^, 383.073 8 [M − H-C_4_H_8_O_4_-C_3_H_6_O_3_]^−^, 353.063 4 [M − H-2C_4_H_8_O_4_]^−^	A	^21^
N6	6.68	Kaempferol-3-*O*-rutinoside	C_27_H_30_O_15_	595.167 1 [M + H]^+^	2.3	287.056 3 [M + H-Rha-Glc]^+^	B/C	^23^
N7	6.72	Kaempferol-3-*O*-(2″,6″-dirhamnosyl)-glucoside	C_33_H_40_O_19_	739.205 8 [M − H]^−^	− 4.5	285.039 1 [C_15_H_10_O_16_]^−^, 284.032 5 [C_15_H_10_O_16_-H]^−^	C	^22^
N8	7.00	Quercetin	C_15_H_10_O_7_	303.049 7 [M + H]^+^	− 0.7	257.045 5 [M + H-H_2_O-CO]^+^, 213.057 1 [M + H-2CO-H_2_O-O]^+^, 166.027 2 [M + H–CO-C_6_H_5_O_2_]^+^	B/D	^24^
N9	7.02	Rutin*	C_27_H_30_O_16_	611.160 2 [M + H]^+^	− 0.8	303.048 2[M + H-Rha-Glc]^+^	B/D	^23^
N10	7.41	Isoquercitrin	C_21_H_20_O_12_	463.086 4 [M − H]^−^	− 3.9	301.034 8 [M − H-Glc]^−^	C/D	^24^
N11	7.61	Ginkgolide J	C_20_H_24_O_10_	425.144 7 [M + H]^+^	− 2.0	407.136 8 [M + H-H_2_O]^+^, 363.144 9 [M + H-H_2_O-CO_2_]^+^	B	^23^
N12	7.66	Neoeriocitrin	C_27_H_32_O_15_	595.164 4 [M − H]^−^	− 4	459.112 3 [M − H-C_8_H_8_O_2_]^−^, 287.055 7 [M − H-Rha-Glc]^−^, 151.003 1 [M − H-Rha-Glc-C_6_H_4_O_2_-CO]^−^	A	^21^
N13	7.89	Ferulic acid*	C_10_H_10_O_4_	193.050 4 [M − H]^−^	− 1.2	178.026 4 [M − H-CH_3_]^−^, 134.037 4 [M − H-CH_3_-CO_2_]^−^	B/D	^24^
N14	7.92	Ginkgolide C	C_20_H_24_O_11_	439.123 3 [M − H]^−^	− 2.9	383.133 0 [M − H-2CO]^−^, 365.124 4 [M − H-2CO-H_2_O]^−^, 321.134 2 [M − H-2CO-H_2_O-CO_2_]^−^, 303.123 5 [M − H-2CO-H_2_O-CO_2_-H_2_O]^−^, 277.145 0 [M − H-2CO-H_2_O-2CO_2_]^−^, 259.133 8 [M − H-2CO-H_2_O-2CO_2_-H_2_O]^−^	B	^23^
N15	8.06	Quercetin-3-*O*-rutinoside	C_27_H_30_O_16_	609.043 6 [M − H]^−^	− 4.1	300.026 5 [M − H-Rha-Glc]^−^, 271.024 2 [M − H-Rha-Glc-CO]^−^, 255.029 2 [M − H-Rha-Glc-CO-O]^−^	C	^23^
N16	8.13	Kaempferol-3-*O*-(2″-*β*-*D*-glucopyranosyl)-α-*L*-rhamnoside	C_27_H_30_O_15_	595.166 1 [M + H]^+^	0.6	449.107 3 [M + H-Rha]^+^, 287.055 3 [M + H-Rha-Glc]^+^	B	^23^
N17	8.57	Kaempferol-4′-*O*-glucoside	C_21_H_20_O_11_	447.092 2 [M − H]^−^	− 2.4	285.040 2[M − H-Glc]^−^	C	^22^
N18	8.98	Rhoifolin	C_27_H_30_O_14_	579.170 2 [M + H]^+^	− 1.1	433.113 9 [M + H-Rha]^+^, 271.058 7 [M + H-Rha-Glc]^+^	A	^21^
N19	9.01	Naringin*	C_27_H_32_O_14_	581.186 0 [M + H]^+^	− 0.9	315.086 5 [M + H-Rha-C_8_H_8_O]^+^, 273.074 3 [M + H-Rha-Glc]^+^, 153.018 6 [M + H-Rha-Glc-C_8_H_8_O]^+^	A/D	^21^
N20	10.66	Meranzin	C_15_H_16_O_4_	261.111 7 [M + H]^+^	− 1.7	189.053 7 [M + H-C_4_H_8_O]^+^, 159.044 5 [M + H-C_4_H_8_O-CH_2_O]^+^, 131.048 7 [M + H-C_4_H_8_O-CH_2_O-CO]^+^	A	^33^
N21	11.22	Isorhamnetin-3-*O*-rutinoside	C_28_H_32_O_16_	625.174 4 [M + H]^+^	− 3.1	479.118 7 [M + H-Rha]^+^, 317.064 1 [M + H-Rha-Glc]^+^	B/C	^23^
N22	11.47	Ginkgolide A*	C_20_H_24_O_9_	409.148 1 [M + H]^+^	− 3.0	391.139 9 [M + H-H_2_O]^+^, 373.130 0 [M + H-2H_2_O]^+^, 355.119 1 [M + H-3H_2_O]^+^, 345.134 8 [M + H-2H_2_O-CO]^+^, 327.124 6 [M + H-3H_2_O-CO]^+^	B	^23^
				407.134 1 [M − H]^−^	− 1.6	351.143 6 [M − H-2CO]^−^, 333.132 1 [M − H-2CO-H_2_O]^−^, 319.144 0 [M − H-2CO_2_]^−^, 307.160 3 [M − H-2CO-CO_2_]^−^, 289.143 3 [M − H-2CO-CO_2_-H_2_O]^−^, 273.148 1 [M − H-2CO_2_-CO-H_2_O]^−^, 245.154 0 [M − H-2CO-H_2_O-2CO_2_]^−^		
N23	11.48	Ginkgolide B*	C_20_H_24_O_10_	425.144 0 [M + H]^+^	− 0.5	407.134 3 [M + H-H_2_O]^+^, 389.124 4 [M + H-2H_2_O]^+^, 371.113 2 [M + H-3H_2_O]^+^, 361.129 4 [M + H-2H_2_O-CO]^+^, 343.119 0 [M + H-3H_2_O-CO]^+^	B	^23^
N24	11.92	Luteolin*	C_15_H_10_O_6_	287.055 5 [M + H]^+^	1.8	153.019 1 [M + H-C_8_H_6_O_2_]^+^	B	^24^
N25	11.94	Morin	C_15_H_10_O_7_	301.033 9 [M − H]^−^	− 4.9	273.040 1[M − H-CO]^−^, 178.998 2, 151.002 9	D	^31^
N26	12.06	Oxypeucedanin hydrate	C_16_H_16_O_6_	305.102 2 [M + H]^+^	0.6	203.034 4 [M + H-C_5_H_10_O_2_]^+^, 175.039 8 [M + H-C_5_H_10_O_2_-CO]^+^, 159.045 2 [M + H-C_5_H_10_O_2_-CO-O]^+^, 147.044 7 [M + H-C_5_H_10_O_2_-2CO]^+^, 131.049 9 [M + H-C_5_H_10_O_2_-2CO-O]^+^	A	^33^
N27	12.92	Quercetin-3-methyl ether	C_16_H_12_O_7_	315.049 6 [M − H]^−^	− 4.5	300.025 3 [M − H-CH_3_]^−^, 271.023 2 [M − H-CH_3_-CO]^−^	D	^24^
N28	14.11	Naringenin*	C_15_H_12_O_5_	273.075 6 [M + H]^+^	− 0.5	153.018 3 [M + H-C_8_H_8_O]^+^, 147.044 5 [M + H-C_6_H_6_O_3_]^+^	A/D	^21^
N29	14.12	Apigenin	C_15_H_10_O_5_	269.044 6 [M − H]^−^	− 3.6	251.036 0 [M − H-H_2_O]^−^, 225.055 1 [M − H-CO_2_]^−^, 151.003 5 [M − H-C_8_H_6_O]^−^, 117.033 9 [M − H-C_7_H_4_O_4_]^−^	A/B/D	^24^
N30	14.28	Pinobanksin	C_15_H_12_O_5_	271.059 9 [M − H]^−^	− 5	253.048 8 [M − H-H_2_O]^−^, 225.055 3 [M − H-H_2_O-CO]^−^	D	^24^
N31	14.42	Kaempferol	C_15_H_10_O_6_	287.055 6 [M + H]^+^	2.1	259.061 0 [M + H–CO]^+^, 231.066 4 [M + H-2CO]^+^	A/B/D	^24^
N32	14.88	Isorhamnetin	C_16_H_12_O_7_	315.049 8 [M − H]^−^	− 3.9	300.025 8 [M − H-CH_3_]^−^	B/D	^24^
N33	15.38	Luteolin-methyl-ether	C_16_H_12_O_6_	299.054 8 [M − H]^−^	− 4.4	284.030 3 [M − H-CH_3_]^−^, 255.028 2	D	^30^
N34	15.46	Kaempferide	C_16_H_12_O_6_	301.069 3 [M + H]^+^	− 4.5	286.046 1 [M + H-CH_3_]^+^, 258.052 7 [M + H-CH_3_-CO]^+^	D	^24^
N35	15.87	Quercetin-dimethyl-ether	C_17_H_14_O_7_	329.065 4 [M − H]^−^	− 3.9	314.040 8 [M − H-CH_3_]^+^, 299.018 0 [M − H-2CH_3_]^+^	D	^24^
N36	16.99	Galangin-5-methyl-ether	C_16_H_12_O_5_	283.059 9 [M − H]^−^	− 4.6	268.036 4 [M − H-CH_3_]^+^, 239.033 2 [M − H-CH_3_-CO_2_]^+^	D	^30^
N37	17.10	Biochanin A	C_16_H_12_O_5_	285.074 8 [M + H]^+^	− 3.3	270.051 0 [M + H-CH_3_]^+^, 242.057 4 [M + H-CH_3_-CO]^+^	D	^29^
N38	17.32	Isomeranzin*	C_15_H_16_O_4_	261.111 3 [M + H]^+^	− 3.2	189.053 9 [M + H-C_4_H_8_O]^+^, 159.044 8 [M + H-C_4_H_8_O-CH_2_O]^+^, 131.049 1 [M + H-C_4_H_8_O-CH_2_O-CO]^+^	A	^33^
N39	18.08	Limonin*	C_26_H_30_O_8_	469.186 3 [M − H]^−^	− 1	423.190 8 [M − H-CO_2_-H_2_O]^−^	A	^33^
N40	18.34	Quercetin-dimethyl-ether	C_17_H_14_O_7_	329.065 5 [M − H]^−^	− 3.7	314.039 9 [M − H-CH_3_]^+^, 299.016 6 [M − H-2CH_3_]^+^, 271.022 4 [M − H-2CH_3_-CO]^+^	D	^24^
N41	18.69	Gypenoside XVII	C_48_H_82_O_18_	991.545 4 [M + HCOO]^−^	− 2.9	945.539 7 [M − H]^−^	C	^22^
N42	19.20	Chrysin*	C_15_H_10_O_4_	253.049 6 [M − H]^−^	− 3.9	209.059 2 [M − H-CO_2_]^−^, 143.049 0 [M − H-C_3_O_2_-C_2_H_2_O]^−^	D	^24^
				255.064 3 [M + H]^+^	− 3.6	153.017 4		
N43	19.58	Caffeic acid benzyl ester	C_16_H_14_O_4_	269.081 9 [M − H]^−^	− 2.4	178.026 7 [M − H-C_7_H_7_]^−^, 134.037 0 [M − H-C_7_H_7_-CO_2_]^−^	D	^24^
N44	19.73	Pinocembrin*	C_15_H_12_O_4_	255.065 3 [M − H]^−^	− 3.8	213.053 8 [M − H-C_2_H_2_O]^−^, 151.002 5	D	^24^
				257.080 7 [M + H]^+^	− 0.7	153.017 3		
N45	19.78	Methoxy-chrysin	C_16_H_12_O_5_	283.059 9 [M − H]^−^	− 4.6	268.034 9 [M-CH_3_]^−^, 239.032 4 [M − H-CO_2_]^−^	D	^24^
N46	19.95	Galangin	C_15_H_10_O_5_	269.044 3 [M − H]^−^	− 4.6	252.042 1 [M − H-H_2_O]^−^, 241.049 6 [M − H –CO]^−^, 213.054 7 [M − H-2CO]^−^	D	^24^
				271.059 8 [M + H]^+^	− 1.3	153.017 7		
N47	20.38	Pinobanksin-3-*O*-acetate	C_17_H_14_O_6_	313.070 5 [M − H]^−^	− 4.0	253.047 6 [M-C_2_H_3_O-H_2_O]^−^, 209.060 2 [M-C_2_H_3_O-H_2_O-CO_2_]^−^	D	^24^
N48	20.57	3*β*,12*β*-20(S)-Trihydroxydammar-24-ene-3-*O*-*β*-*D*-glucopyranosyl-20-*O*-[*β*-*D*-6-*O*-acetylglucopyranosyl-(1–2)-*β*-*D*-glucopyranoside]	C_50_H_84_O_19_	1033.556 0 [M + HCOO]^−^	− 2.8	987.551 5 [M − H]^−^, 945.538 6 [M − H-C_2_H_2_O]^−^	C	^22^
N49	20.64	Phenethyl caffeate	C_17_H_16_O_4_	283.096 6 [M − H]^−^	− 3.5	179.033 6 [M-C_8_H_9_]^−^, 135.043 8 [M-C_8_H_9_-CO_2_]^−^	D	^24^
N50	20.90	Acacetin	C_16_H_12_O_5_	283.060 0 [M − H]^−^	− 4.1	268.034 9[M − H-CH_3_]^−^, 239.032 3 [M − H-CH_3_-CO]^−^, 211.037 5	D	^28^
N51	21.38	Eupatilin	C_18_H_16_O_7_	343.081 9 [M − H]^−^	− 1.4	328.057 0 [M − H-CH_3_]^−^, 313.034 1 [M − H-2CH_3_]^−^, 285.039 3 [M − H-2CH_3_-CO]^−^	D	^28^
N52	21.54	Asiatic acid	C_30_H_48_O_5_	487.342 2 [M − H]^−^	− 1.5	423.030 2 [M − H CO_2_H-H_2_O-H]^−^	D	^27^
N53	21.72	Gypenoside LXXIV	C_42_H_72_O_14_	845.489 4 [M + HCOO]^−^	− 1.2	799.482 6 [M − H]^−^, 637.431 1 [M − H-Glc]^−^, 475.379 9 [M − H-2Glc]^−^	C	^22^
N54	22.19	Caffeic acid cinnamyl ester	C_18_H_16_O_4_	295.096 3 [M − H]^−^	− 4.3	178.026 2 [M − H-C_9_H_9_]^−^, 134.035 9 [M − H-C_9_H_9_-CO_2_]^−^	D	^24^
N55	22.67	Pinobanksin-3-*O*-propionate	C_18_H_16_O_6_	327.085 9 [M − H]^−^	− 4.6	253.047 9 [M-C_3_H_5_O-H_2_O]^−^, 209.060 2 [M-C_3_H_5_O-H_2_O-CO_2_]^−^, 185.060 5 [M-C_3_H_5_O-H_2_O-C_3_O_2_]^−^, 181.065 8 [M-C_3_H_5_O-H_2_O-CO_2_-CO]^−^, 165.070 9 [M-C_3_H_5_O-H_2_O-2CO_2_]^−^	D	^24^
N56	23.13	Ginsenoside-F_2_	C_42_H_72_O_13_	829.492 8 [M + HCOO]^−^	− 3.2	783.488 2 [M − H]^−^, 621.438 0 [M − H-Glc]^−^, 459.382 0 [M − H-2Glc]^−^	C	^22^
N57	23.44	Ginsenoside Rg3	C_42_H_72_O_13_	829.492 5 [M + HCOO]^−^	− 3.6	783.486 5 [M − H]^−^, 621.434 6 [M − H-Glc]^−^, 459.386 3 [M − H-2Glc]^−^	C	^26^
N58	24.61	Pinobanksin-3-*O*-butyrate	C_19_H_18_O_6_	341.101 9 [M − H]^−^	− 3.4	253.048 1 [M-C_4_H_7_O-H_2_O]^−^, 209.060 6 [M-C_4_H_7_O-H_2_O-CO_2_]^−^, 143.050 1 [M-C_4_H_7_O-H_2_O-C_3_O_2_-C_2_H_2_O]^−^	D	^24^
N59	24.72	Chrysin-5-methyl-ether	C_16_H_12_O_4_	269.079 9 [M + H]^+^	− 3.5	254.056 8 [M + H-CH_3_]^+^, 226.061 2	D	^24^
N60	24.78	Pinobanksin-3-*O*-pentenoate	C_20_H_18_O_6_	353.101 4 [M − H]^−^	− 4.7	271.060 4 [M-C_5_H_7_O]^−^, 253.048 6 [M-C_5_H_7_O-H_2_O]^−^	D	^24^
N61	25.00	Damulin B	C_42_H_70_O_13_	827.476 1 [M + HCOO]^−^	− 4.5	781.475 9 [M − H]^−^, 619.425 1 [M − H-Glc]^−^	C	^22^
N62	26.32	Pinobanksin-3-*O*-pentenoateor-2-methylbutyrate	C_20_H_20_O_6_	355.117 3 [M − H]^−^	− 4.0	253.047 8 [M-C_5_H_9_O-H_2_O]^−^	D	^24^
N63	26.62	Pinobanksin-3-*O*-hexenoate	C_21_H_20_O_6_	367.117 0 [M − H]^−^	− 4.7	271.060 2 [M-C_6_H_9_O]^−^, 253.049 3 [M-C_6_H_9_O-H_2_O]^−^	D	^24^
N64	26.87	Pinobanksin-3-*O*-phenylpropionate	C_24_H_20_O_6_	403.116 8 [M − H]^−^	− 4.7	253.049 4 [M-C_9_H_9_O-H_2_O]^−^	D	^24^
N65	27.84	Pinobanksin-3-*O*-hexanoate	C_21_H_22_O_6_	369.132 6 [M − H]^−^	− 4.8	253.048 3 [M-C_6_H_11_O-H_2_O]^−^	D	^24^
N66	29.99	Auraptene	C_19_H_22_O_3_	299.164 7 [M + H]^+^	1.8	163.040 0 [M + H-C_10_H_16_]^+^, 145.103 8 [M + H-C_10_H_16_-H_2_O]^+^, 135.042 6 [M + H-C_10_H_16_-CO]^+^, 107.050 0 [M + H − C_10_H_16_ − 2CO]^+^	A	^33^

Glc: glucose; Rha: rhamnose.

A. *Citri Grandis Exocarpium.*

B. *Ginkgo Folium.*

C. *Gynostemma pentaphyllum.*

D. Propolis.

*Identified by comparison with reference standards (Supplementary Fig. S1).

### Analysis of chemical constituent in YLTZC by UPLC-Q-TOF–MS/MS

#### Flavonoids

**Figure 2:**
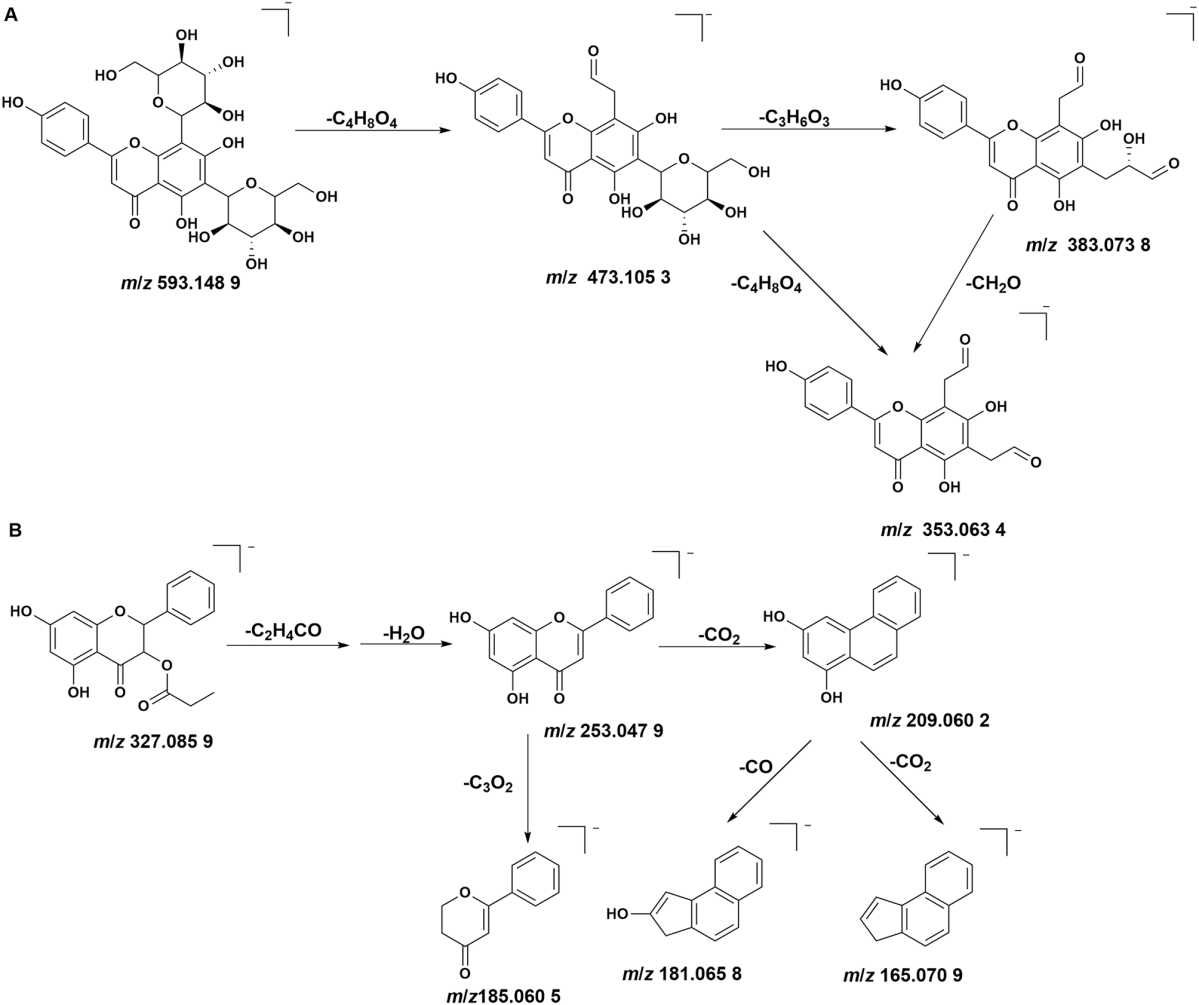
The fragmentation pattern of vicenin-2 (**A**) and pinobanksin-3-*O*-propionate (**B**).

A total of 42 flavonoids (peak 5–10, 12, 15–19, 21, 24–25, 27–37, 40, 42, 44–47, 50–51, 55, 58–60, 62–65) were identified from YLTZC, and the flavonoids mainly came from propolis, *Citri Grandis Exocarpium* and *Ginkgo Folium*, in the form of free or glycoside, and etc. The identification process was analyzed by taking peak 5, peak 12, and 55 as examples. In negative ion mode, peak 5 had [M − H]^−^ ions at *m*/*z* 593.148 9, its MS/MS fragment ions at* m*/*z* 473.105 3 [M − H-C_4_H_8_O_4_]^−^, *m*/*z* 383.073 8 [M − H-C_4_H_8_O_4_-C_3_H_6_O_3_]^−^, *m*/*z* 353.063 4 [M − H-2C_4_H_8_O_4_]^−^, and *m*/*z* 353.063 4 [M − H-C_4_H_8_O_4_-C_3_H_6_O_3_-CH_2_O]^−^ were further pointed out by comparing with the reference standard and literature^21^. Thus, peak 5 was deduced as vicenin-2, the detailed fragmentation pathway of peak 5 is shown in Fig. 2A. Peak 55 showed at *m*/*z* 327.085 9 [M − H]^−^, with *m*/*z* 253.047 9 [M-C_3_H_5_O-H_2_O]^−^, *m*/*z* 209.060 2 [M-C_3_H_5_O-H_2_O-CO_2_]^−^, *m*/*z* 181.065 8 [M-C_3_H_5_O-H_2_O-CO_2_-CO]^−^, *m*/*z* 185.060 5 [M-C_3_H_5_O-H_2_O-C_3_O_2_]^−^, *m*/*z* 165.070 9 [M-C_3_H_5_O-H_2_O-2CO_2_]^−^ being the main fragment ions in negative ion mode. Based on the reports in literature^24^, peak 55 was deduced as pinobanksin-3-*O*-propionate, the detailed fragmentation pathway of peak 55 is shown in Fig. 2B.

#### Saponins

**Figure 3:**
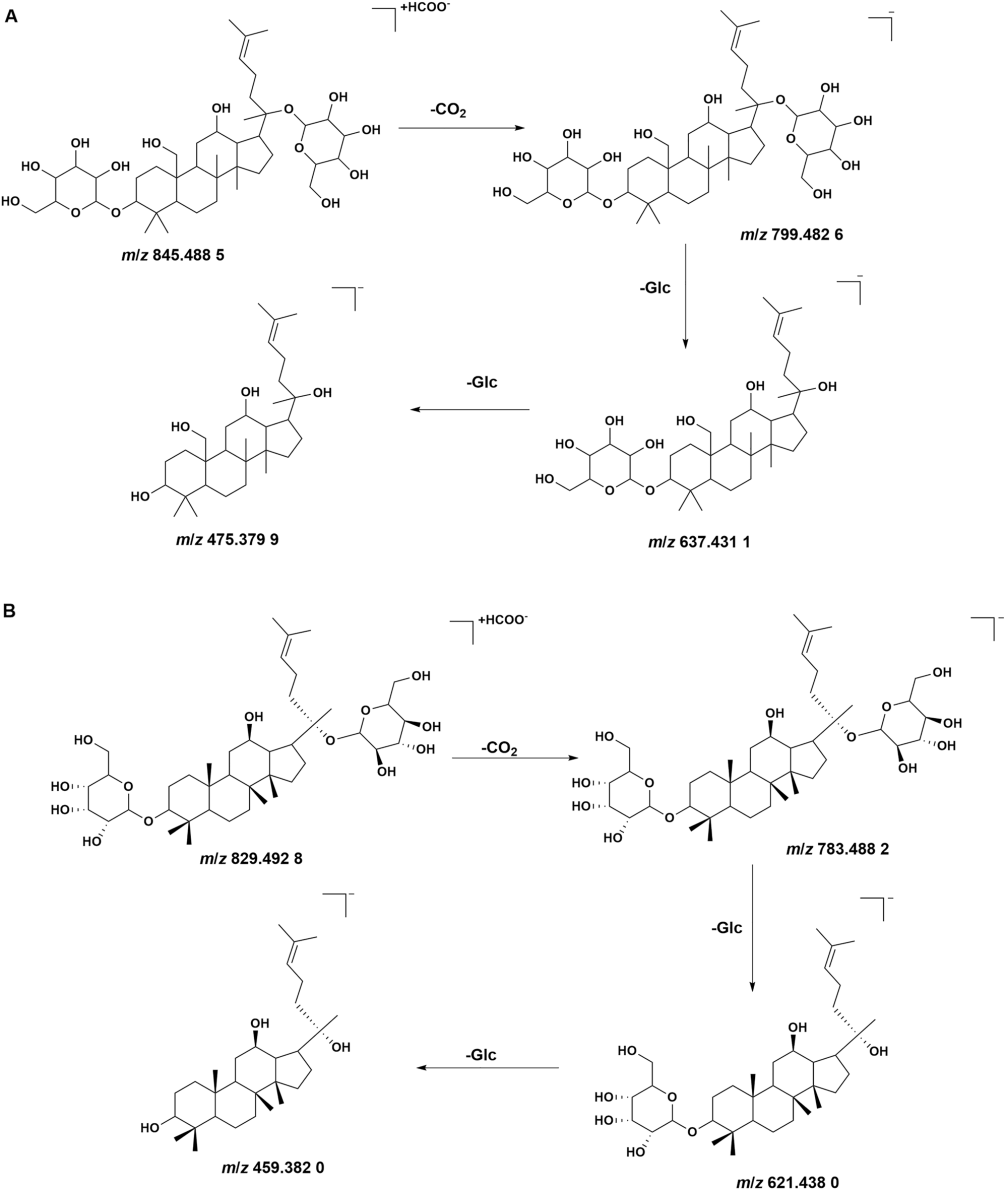
The fragmentation pattern of gypenoside LXXIV (**A**) and ginsenoside-F_2_ (**B**).

Six saponins (peak 41, 48, 53, 56–57, 61) were detected from YLTZC, all from *Gynostemma pentaphyllum*. These compounds mainly exist in the form of anion [M + HCOO]^−^, and the cracking law is mainly manifested as loss of –C_2_H_2_O, –C_4_H_4_O and glycosidic. For instance, the mass loss of 162 Da, 146 Da and 132 Da represents the loss of glucose (Glc), rhamnose (Rha), and xylose (Xyl), respectively. Peak 53 had [M − H]^−^ and [M + HCOO]^−^ ion at *m*/*z* 799.482 6 and *m*/*z* 845.489 4 in negative ion mode. The fragment ions at *m*/*z* 637.431 1 [M − H-Glc]^−^ and *m*/*z* 475.379 9 [M − H-2Glc]^−^ were formed by the [M − H]^−^ ion following succession to lose of Glc residue. Based on the reports in literature, it was tentatively characterized as gypenoside LXXIV, the detailed fragmentation pathway of peak 53 is shown in Fig. 3A. Peak 56 had [M − H]^−^ and [M + HCOO]^−^ ion at *m*/*z* 783.488 2 and *m*/*z* 829.492 8 in negative ion mode. The fragment ions at *m*/*z* 621.438 0 [M − H-Glc]^−^ and *m*/*z* 459.382 0 [M − H-2Glc]^−^ were formed by the [M − H]^−^ ion following succession to eliminate Glc residue. Hence, it was tentatively characterized as ginsenoside-F_2_, the detailed fragmentation pattern of ginsenoside-F_2_ is shown in Fig. 3B^22^.

#### Coumarins

**Figure 4:**
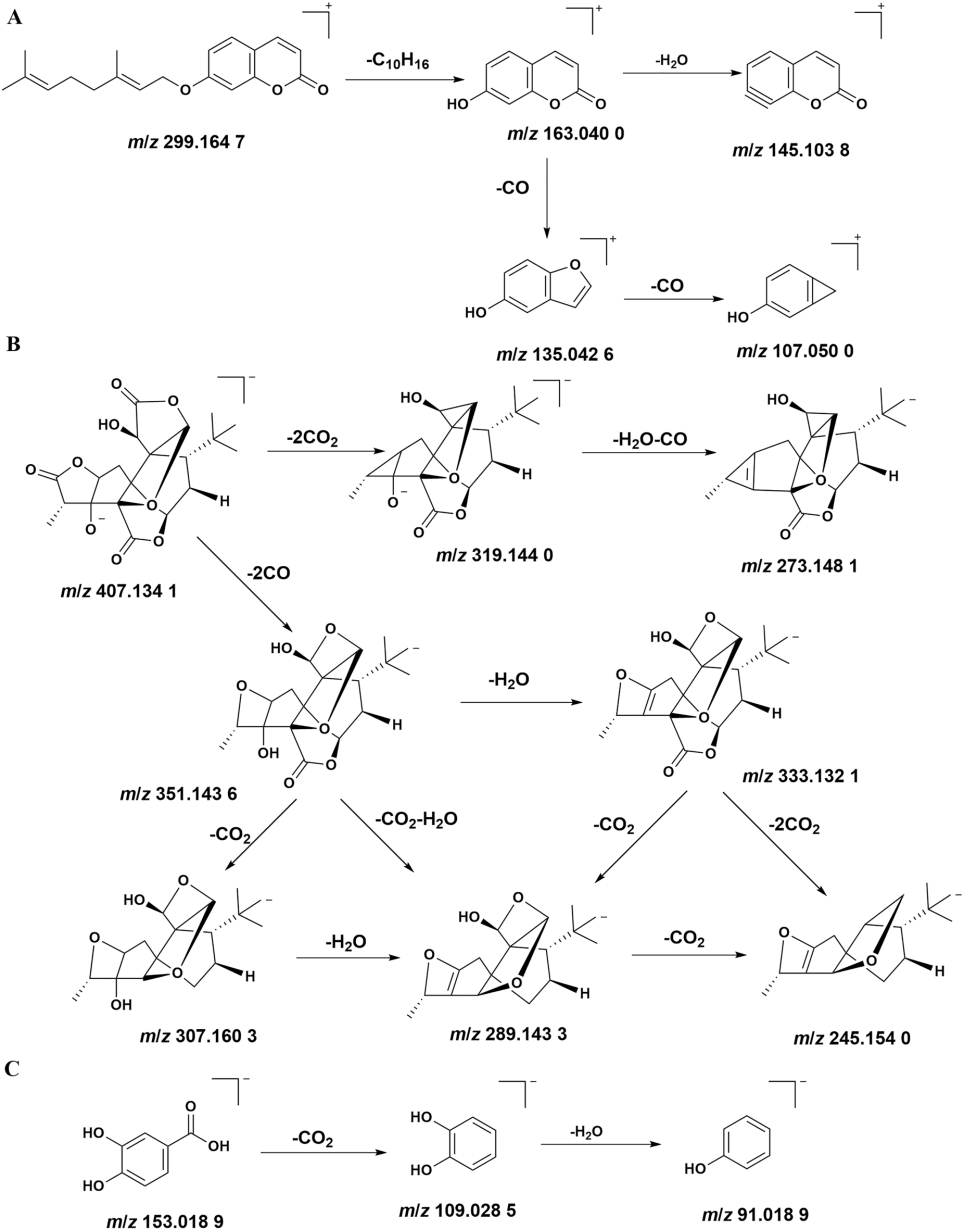
The fragmentation pattern of auraptene (**A**), ginkgolide A (**B**), and protocatechuic acid (**C**).

Four coumarins (peak 20, 26, 38, 66) were identified from YLTZC, all of which were derived from *Citri Grandis Exocarpium*, and most coumarins have stronger response in the positive ion mode. Peak 38 exhibited [M + H]^+^ ion at *m*/*z* 261.111 3M + H]^+^, with *m*/*z* 189.053 9 [M + H-C_4_H_8_O]^+^, *m*/*z* 159.044 8 [M + H-C_4_H_8_O-CH_2_O]^+^, *m*/*z* 131.049 1 [M + H-C_4_H_8_O-CH_2_O-CO]^+^ as the fragment ions in positive mode, by comparing with the reference standard, peak 38 was deduced as isomeranzin. Peak 20, and 38 are a group of isomers, based on the reports in literature, peak 20 was deduced as meranzin. In positive ion mode, peak 66 had [M + H]^+^ ions at *m*/*z* 299.164 7, and the main fragment ions at *m*/*z* 163.040 0 [M + H-C_10_H_16_]^+^, *m*/*z* 145.103 8 [M + H-C_10_H_16_-H_2_O]^+^, *m*/*z* 135.042 6 [M + H-C_10_H_16_-CO]^+^, *m*/*z* 107.050 0 [M + H-C_10_H_16_-2CO]^+^, by based on the reports in literature^33^, peak 66 was inferred as auraptene. The detailed mass fragment pathway of peak 66 is shown in Fig. 4A.

#### Lactones

Four lactones (peak 11, 14, 22–23) were detected from YLTZC, all of which were derived from *Ginkgo Folium*. Ginkgolides have similar cleavage pathways, and the typical cleavage pathways is the opening of the lactone ring, with continuous loss of CO, CO_2_, and H_2_O. Peak 22, and peak 23 were identified as ginkgolide A, and ginkgolide B by comparing with reference standards. Taking peak 22 as an example in detail to illuminate the MS fragmentation pattern of these diterpenoid lactones^23^. Peak 22 had [M − H]^−^ ions at *m*/*z* 407.134 1 in negative ion mode. And then [M − H]^−^ ion continuous loss of CO, CO_2_, and loss of H_2_O to form *m*/*z* 351.143 6 [M − H-2CO]^−^,* m*/*z* 333.132 1 [M − H-2CO-H_2_O]^−^,* m*/*z* 319.144 0 [M − H-2CO_2_]^−^,* m*/*z* 307.160 3 [M − H-2CO-CO_2_]^−^,* m*/*z* 289.143 3 [M − H-2CO-CO_2_-H_2_O]^−^,* m*/*z* 273.148 1 [M − H-2CO_2_-CO-H_2_O]^−^,* m*/*z* 245.154 0 [M − H-2CO-H_2_O-2CO_2_]^−^. Therefore, the compound was identified as ginkgolide A. The detailed mass fragment pathway of peak 22 is shown in Fig. 4B.

#### Organic acids

A total of nine organic acids (peak 1–4, 13, 43, 49, 52, 54) were identified. Taking peak 2 as an example, peak 2 exhibited [M − H]^−^ ion at *m*/*z* 153.018 9 [M − H]^−^, with *m*/*z* 109.028 5 [M − H-CO_2_]^−^,* m*/*z* 91.018 9 [M − H-CO_2_-H_2_O]^−^ as the fragment ions in negative mode, by comparing with the reference standard^24^, peak 2 was deduced as protocatechuic acid, the detailed mass fragment pathway of peak 2 is shown in Fig. 4C.

### Network pharmacology analysis

#### Collection and screening of potential constituents and targets

We performed an in-depth assessment of the absorption, distribution, metabolism, and excretion-related properties of 66 constituents in YLTZC using the online tool SwissADME. A total of 38 constituents in YLTZC were screened from the SwissADME tool (Table 3). The YLTZC-related targets and HLP-related targets were acquired by the SwissTargetPrediction, GeneCards, and CTD database, respectively. A total of 568 YLTZC-related and 179 HLP-related targets were acquired after searching, integrating and deduplicating steps.

**Table 3:** Active ingredients list of YLTZC.

Name	Compound	Formula	Pharmac-okinetics	Druglikeness				
			GI absorption	Lipinski	Ghose	Veber	Egan	Muegge
N1	Gallic acid	C_7_H_6_O_5_	High	Yes	No	Yes	Yes	No
N2	Protocatechuic acid	C_7_H_6_O_4_	High	Yes	No	Yes	Yes	No
N3	4-Hydroxycinnamic acid	C_9_H_8_O_3_	High	Yes	Yes	Yes	Yes	No
N4	Vanillic acid	C_8_H_8_O_4_	High	Yes	Yes	Yes	Yes	No
N8	Quercetin	C_15_H_10_O_7_	High	Yes	Yes	Yes	Yes	Yes
N13	Ferulic acid	C_10_H_10_O_4_	High	Yes	Yes	Yes	Yes	No
N20	Meranzin	C_15_H_16_O_4_	High	Yes	Yes	Yes	Yes	Yes
N22	Ginkgolide A	C_20_H_24_O_9_	High	Yes	Yes	Yes	Yes	Yes
N24	Luteolin	C_15_H_10_O_6_	High	Yes	Yes	Yes	Yes	Yes
N25	Morin	C_15_H_10_O_7_	High	Yes	Yes	Yes	Yes	Yes
N27	Quercetin-3-methyl ether	C_16_H_12_O_7_	High	Yes	Yes	Yes	Yes	Yes
N28	Apigenin	C_15_H_10_O_5_	High	Yes	Yes	Yes	Yes	Yes
N29	Naringenin	C_15_H_12_O_5_	High	Yes	Yes	Yes	Yes	Yes
N30	Pinobanksin	C_15_H_12_O_5_	High	Yes	Yes	Yes	Yes	Yes
N31	Kaempferol	C_15_H_10_O_6_	High	Yes	Yes	Yes	Yes	Yes
N32	Isorhamnetin	C_16_H_12_O_7_	High	Yes	Yes	Yes	Yes	Yes
N33	Luteolin-methyl-ether	C_16_H_12_O_6_	High	Yes	Yes	Yes	Yes	Yes
N34	Kaempferide	C_16_H_12_O_6_	High	Yes	Yes	Yes	Yes	Yes
N35	Quercetin-dimethyl-ether	C_17_H_14_O_7_	High	Yes	Yes	Yes	Yes	Yes
N36	Galangin-5-methyl-ether	C_16_H_12_O_5_	High	Yes	Yes	Yes	Yes	Yes
N37	Biochanin A	C_16_H_12_O_5_	High	Yes	Yes	Yes	Yes	Yes
N38	Isomeranzin	C_15_H_16_O_4_	High	Yes	Yes	Yes	Yes	Yes
N39	Limonin	C_26_H_30_O_8_	High	Yes	Yes	Yes	Yes	Yes
N42	Chrysin	C_15_H_10_O_4_	High	Yes	Yes	Yes	Yes	Yes
N43	Caffeic acid benzyl ester	C_16_H_14_O_4_	High	Yes	Yes	Yes	Yes	Yes
N44	Pinocembrin	C_15_H_12_O_4_	High	Yes	Yes	Yes	Yes	Yes
N45	Methoxy-chrysin	C_16_H_12_O_5_	High	Yes	Yes	Yes	Yes	Yes
N46	Galangin	C_15_H_10_O_5_	High	Yes	Yes	Yes	Yes	Yes
N47	Pinobanksin-3-*O*-acetate	C_17_H_14_O_6_	High	Yes	Yes	Yes	Yes	Yes
N49	Phenethyl caffeate	C_17_H_16_O_4_	High	Yes	Yes	Yes	Yes	Yes
N50	Acacetin	C_16_H_12_O_5_	High	Yes	Yes	Yes	Yes	Yes
N51	Eupatilin	C_18_H_16_O_7_	High	Yes	Yes	Yes	Yes	Yes
N52	Asiatic acid	C_30_H_48_O_5_	High	Yes	No	Yes	Yes	No
N53	Gypenoside LXXIV	C_42_H_72_O_14_	High	Yes	No	Yes	Yes	No
N54	Caffeic acid cinnamyl ester	C_18_H_16_O_4_	High	Yes	Yes	Yes	Yes	Yes
N56	Ginsenoside-F_2_	C_42_H_72_O_13_	High	Yes	No	Yes	Yes	No
N59	Chrysin-5-methyl-ether	C_16_H_12_O_4_	High	Yes	Yes	Yes	Yes	Yes
N66	Auraptene	C_19_H_22_O_3_	High	Yes	Yes	Yes	Yes	No

#### Construction of C-T-D and PPI network

**Figure 5:**
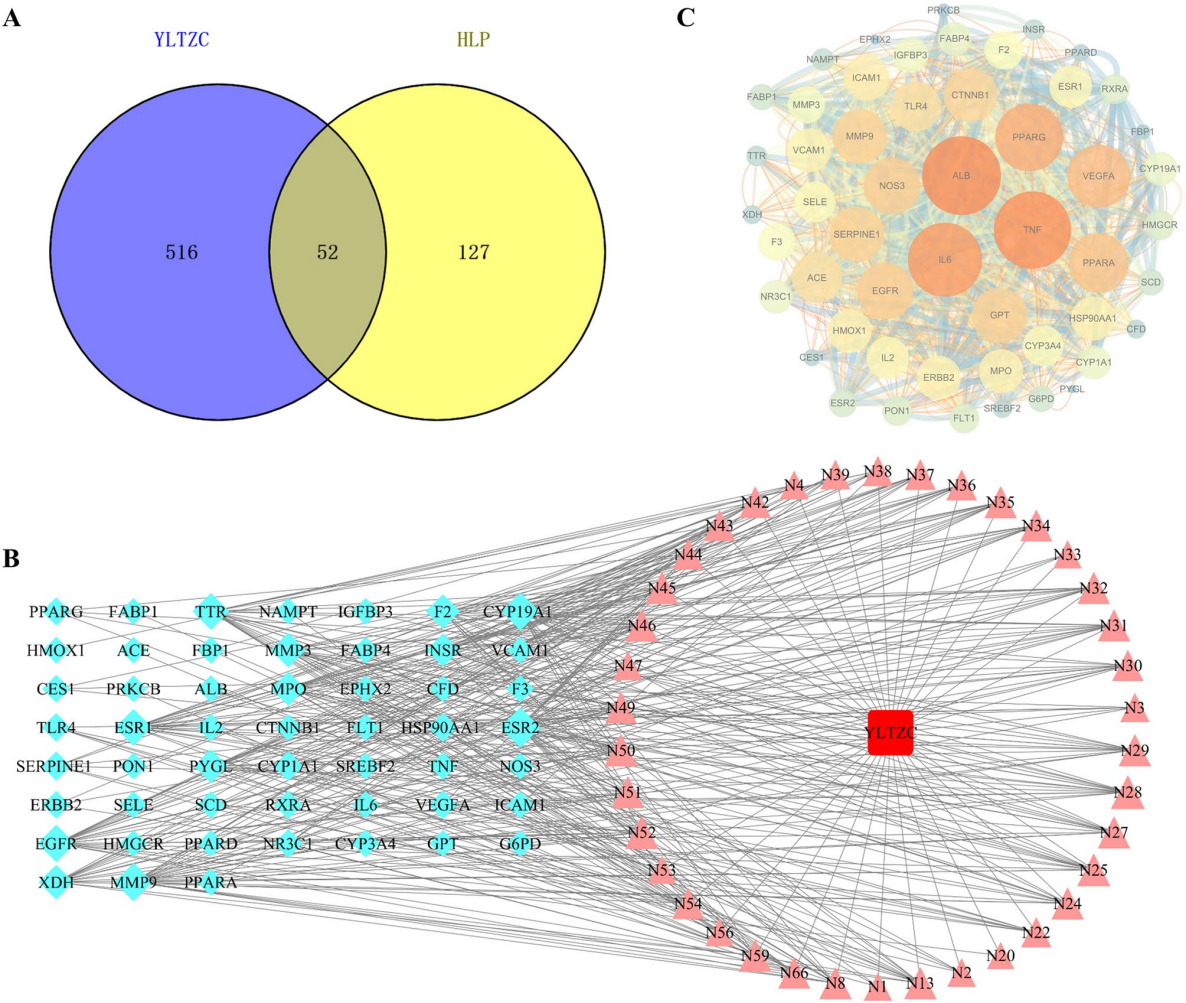
(**A**) Venn diagram of related targets of YLTZC and HLP. (**B**) C-T-D network of YLTZC (Light blue diamond represents target; Rosy triangle represents ingredient in YLTZC. It’s a positive proportional relationship that between the node size and the degree). (**C**) The PPI network is based on the targets of YLTZC. Nodes represent different proteins. Edges represent the association between proteins. The bigger size and brighter color represent higher DC value.

We used Venny 2.1 to obtain the therapeutic targets of YLTZC against HLP (Fig. 5A). 52 overlapping targets were obtained. The C-T-D network contains 91 nodes and 368 edges, including 38 active constituents, and 52 common targets (Fig. 5B). In network analysis, edges represent the interactions between different nodes, and the degree value is decided by the number of connections between a node and other nodes. The higher the degree value, the more significant it represents. According to the degree analysis, the top three compounds were naringenin (N28), asiatic acid (N52), and morin (N25), which may be the active ingredients of YLTZC in alleviating HLP. In addition, a PPI network was constructed based on common targets. As a result, 52 nodes and 996 edges were involved in this network (Fig. 5C). According to the degree analysis, the top 5 potential targets of DC values were selected as the core target, such as ALB, TNF, IL6, PPARG, and VEGFA.

#### Enrichment analysis and C–T–P network

**Figure 6:**
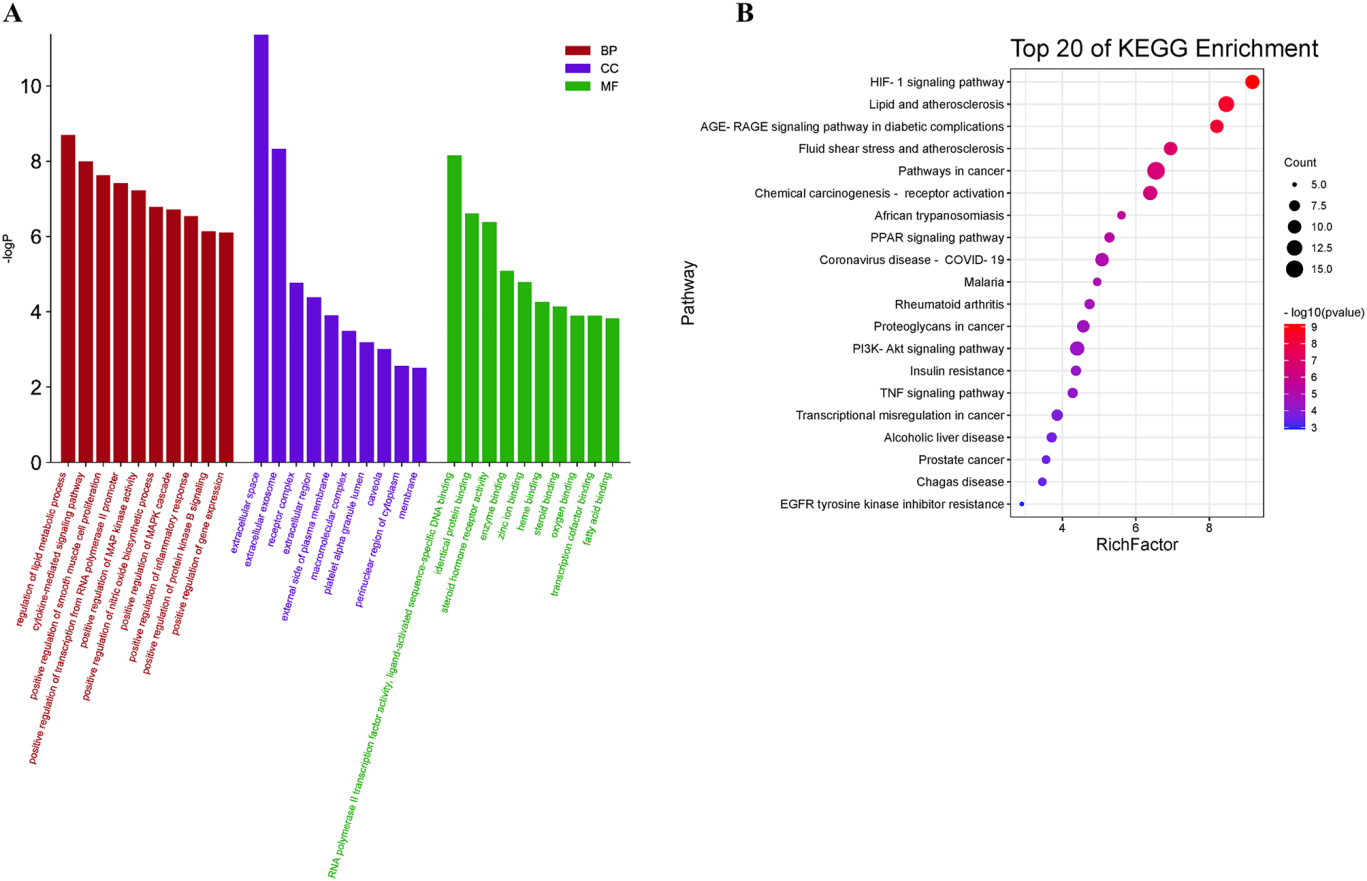
GO (**A**) and KEGG pathway (**B**) enrichment analysis of results for HLP treatment of YLTZC.

**Figure 7:**
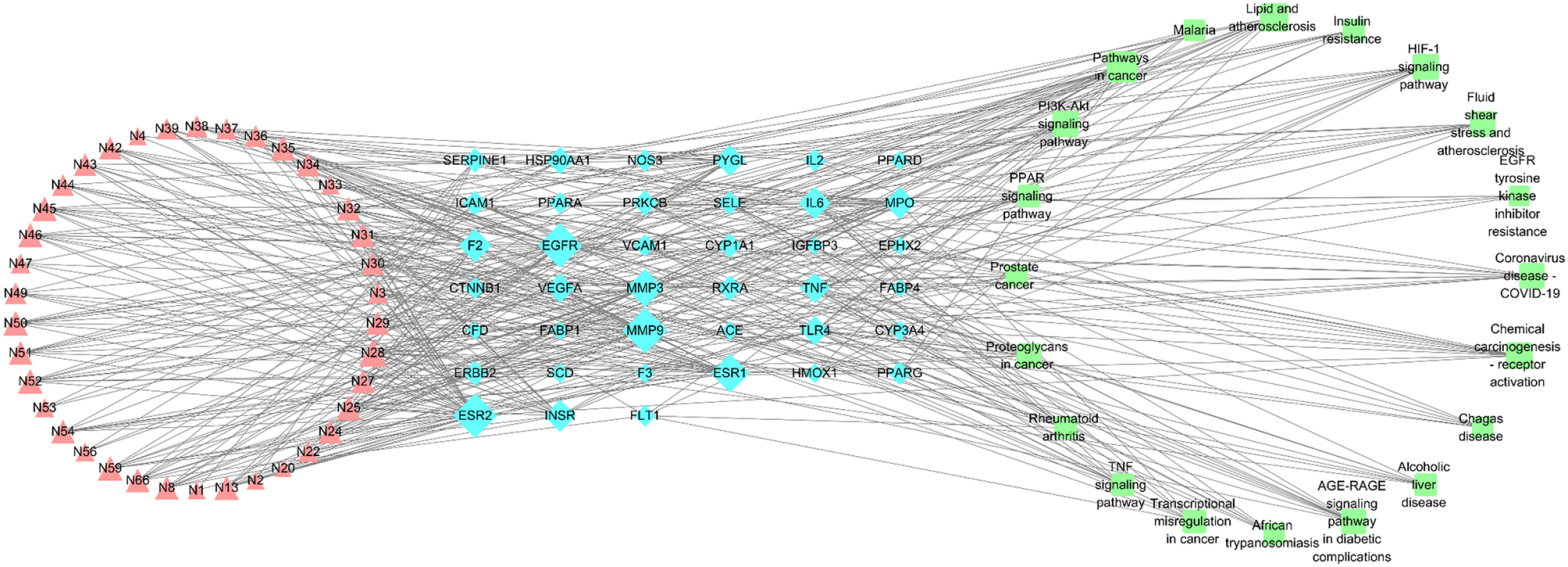
C-T-P network for HLP treatment of YLTZC (Rosy triangle represents ingredients in YLTZC; Light blue diamond represents target; Light green round rectangle represents pathway. It’s a positive proportional relationship that between the node size and the degree).

Gene Ontology (GO) and KEGG pathway enrichment analysis were undertaken on the 52 common targets mentioned above using the DAVID 6.8 database. The top 10 GO enrichment analysis results listed in BP, MF and CC are shown in Fig. 6A. Among them, the BP entries, including extracellular space regulation of lipid metabolic process, positive regulation of smooth muscle cell proliferation, MAPK cascade, inflammatory response, etc. The CC entries, including extracellular space, extracellular exosome, external side of plasma membrane, caveola, membrane, etc. Simultaneously, the MF entries, including RNA polymerase II transcription factor activity, ligand-activated sequence-specific DNA binding, identical protein binding, enzyme binding, steroid binding, oxygen binding, fatty acid binding, etc. The common targets of YLTZC in treating HLP were mainly associated with signaling pathways, such as HIF-1 signaling pathway, AGE-RAGE signaling pathway in diabetic complications, PPAR signaling pathway, PI3K-Akt signaling pathway, Insulin resistance (IR), TNF signaling pathway, etc. The top 20 (*p* < 0.05) KEGG pathways were shown in Fig. 6B. A C–T–P network was constructed to further study the relationship between ingredients, targets and pathways. As shown in Fig. 7, containing 39 targets, 38 active constituents and 20 KEGG pathways was established, including 97 nodes and 422 edges. With topological analysis, we selected two compounds with the highest degree value, naringenin (DC = 12) and ferulic acid (DC = 9) as the core active constituents in YLTZC.

#### Core target molecular docking

**Figure 8:**
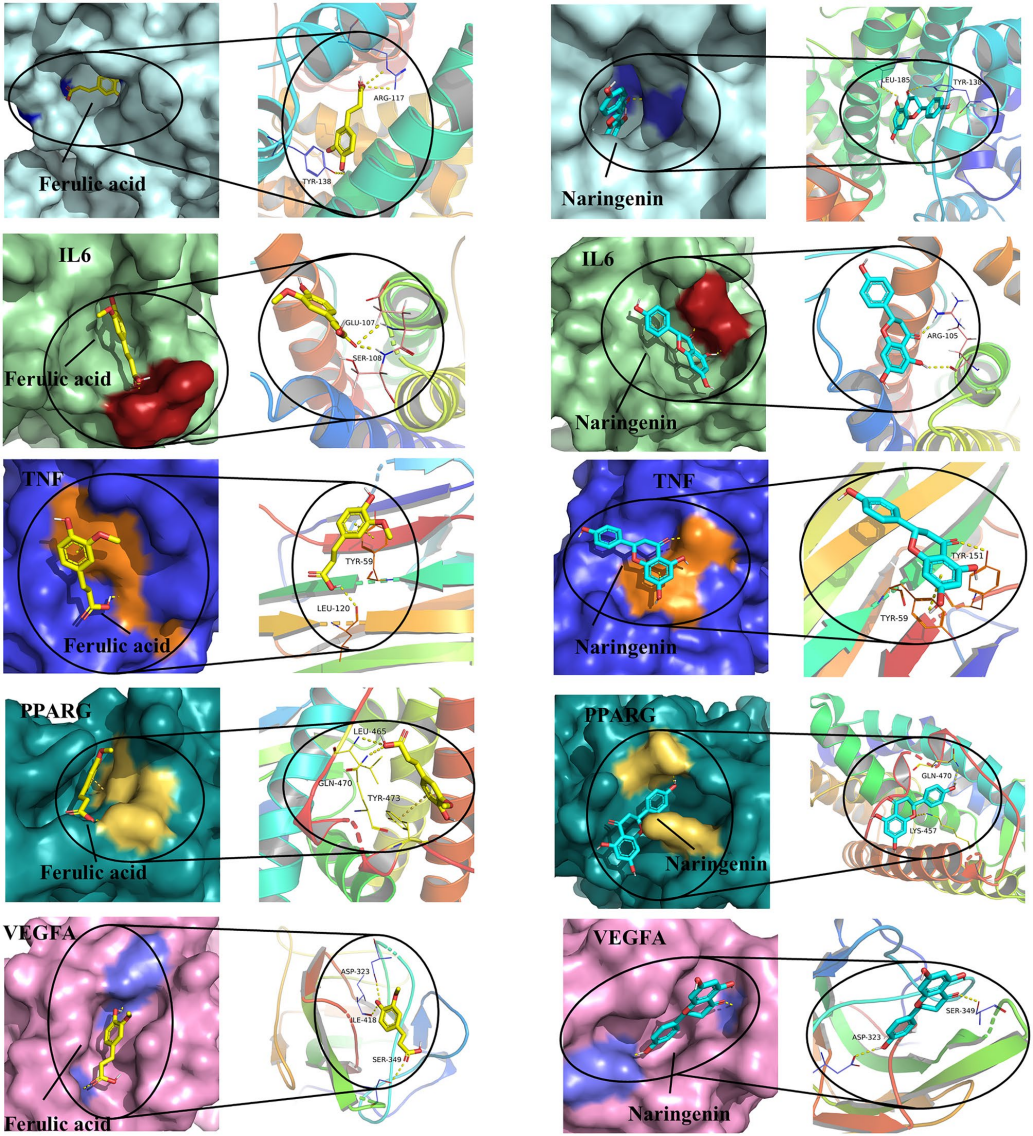
Docking patterns of core targets (ALB, TNF, IL6, PPARG, and VEGFA) and core active constituents (naringenin, and ferulic acid) of YLTZC.

Based on the PPI and C–T–P network, we selected molecular docking between the 2 core active constituents and the top 5 core targets. The docking score and local structure of the results are presented in Table 4 and Fig. 8. The binding sites on the protein surface are indicated by different colors and the hydrogen bonds are shown as dashed lines. Furthermore, the chemical constituent acts on multiple amino acid residues suggesting the multi-target property of TCM preparation. The results showed that most of the affinity energies of the core active constituents docking with core proteins were less than − 5.0 kcal/mol, indicating stable binding^34^. Naringenin and ferulic acid had good affinity with the core targets, which was consistent with the results of literature reports, indicating that naringenin and ferulic acid have good anti-HLP effects. Based on these data, we suggested that the core active constituents have a good affinity for the core targets, which also demonstrated that YLTZC exerted its efficacy through multi-target combination.

**Table 4:** Molecular docking parameter table.

Target protein	Docking parameters	Naringenin	Ferulic acid
ALB	Binding energy/kJ mol^−1^	− 8.3	− 6.1
(4l9k)	Participating amino acid residues (H-Bonds/π-Interactions)	H-donor LEU-185, TYR-138	H-donor ARG-117, TYR-138
TNF	Binding energy/kJ mol^−1^	− 5.8	− 5.0
(2az5)	Participating amino acid residues (H-Bonds/π-Interactions)	H-donor TYR 151/pi-pi TYR 59	H-donor LEU 120/pi-pi TYR 59
IL6	Binding energy/kJ mol^−1^	− 5.8	− 5.1
(1il6)	Participating amino acid residues (H-Bonds/π-Interactions)	H-donor ARG 105	H-donor GLU 107, SER 108
PPARG	Binding energy/kJ mol^−1^	− 6.6	− 5.7
(2fvj)	Participating amino acid residues (H-Bonds/π-Interactions)	H-donor LYS 457, GLN 470	H-donor LEU 465, GLN 470, TYR 473
VEGFA	Binding energy/kJ mol^−1^	− 7.8	− 6.8
(5n21)	Participating amino acid residues (H-Bonds/π-Interactions)	H-donor ASP 323, SER 349	H-donor ASP 323, SER 349, ILE 418

### Experimental evaluation

#### Active ingredients of YLTZC modulated serum lipid levels

**Figure 9:**
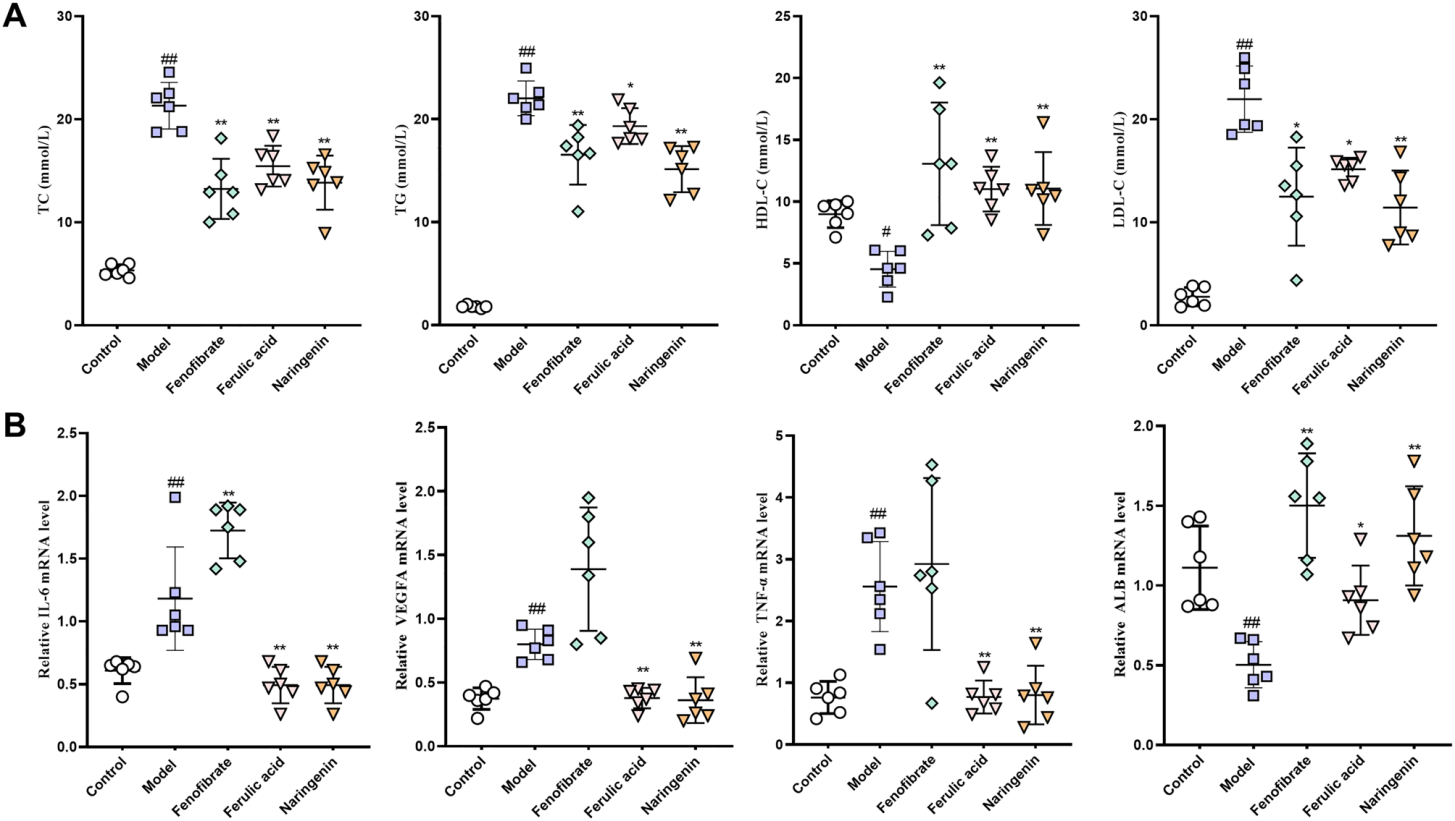
Effect of YLTZC on serum and liver in triton WR-1339-induced HLP mice. (**A**) Biochemical analyses of serum TC, TG, HDL-c and LDL-c. (**B**) The expression of IL-6, VEGFA, TNF-α and ALB mRNA level. Values are mean ± SD, n = 6. ^#^*p* < 0.05, ^##^*p* < 0.01 vs control group; *p < 0.05, **p < 0.01 vs model group.

Firstly, we investigate ferulic acid and naringenin on serum lipid levels in acute hyperlipidemia in triton WR-1339-induced mice. As shown in Fig. 9A, the levels of serum TC, TG, and LDL-C (*p* < 0.01) were significantly increased in model group compared with the control group, and the levels of HDL-C (*p* < 0.05) was decreased. Compared with the model group, the levels of serum TC, TG, and LDL-c (*p* < 0.05, *p* < 0.01) were reduced in the naringenin and ferulic acid group, and the levels of serum HDL-C (*p* < 0.01) was significantly increased. These results suggested that naringenin and ferulic acid from YLTZC have good effect on improving blood lipid levels.

#### Effect of active ingredients of YLTZC on the expression of mRNA of key targets

To clarify the molecular mechanism of YLTZC treatment for HLP, the mRNA expressions of the targets predicted above were measured by RT-PCR. As shown in Fig. 9B, compared to the control group, the triton WR-1339-induced group showed significantly decreased ALB mRNA expression and increased IL6, TNF and VEGFA mRNA expression (all *p* < 0.01), while naringenin and ferulic acid treatment markedly reversed these key targets. As expected, naringenin and ferulic acid treatment significantly increased ALB mRNA expression and decreased IL6, TNF and VEGFA mRNA expression compared to the model group.

In conclusion, these results demonstrated that naringenin and ferulic acid from YLTZC may modulate the mechanism of angiogenesis and inhibiting inflammatory responses by regulating the expression of ALB, IL-6, TNF-α, and VEGFA to alleviate HLP.

## Discussion

HLP is one of the leading risk factors for the development and progression of cardiovascular diseases, characterized by elevated TC, TG, LDL-c, and decreased HDL-c^1^. In our previous study, YLTZC showed a strong hypolipidemic effect, the results showed that YLTZC significantly decreased the levels of serum TC, TG, and LDL-c, and enhanced the level of serum HDL-c in HLP mice^5–7^. However, previous studies provided clues for the current study, but were not comprehensive, and relevant studies on the material basis and related mechanisms of action of YLTZC in the treatment of HLP are still lacking. This limits the further clinical studies and quality control and evaluation of YLTZC. Therefore, this study fills these shortages by developing a comprehensive research method combining chemical profile with network pharmacology, molecular docking, and experimental verification.

In recent years, along with the generalization of systems biology, network pharmacology has become an important method to clarify the potential mechanisms of multiple constituents, targets, and pathways of TCM. In this study, the chemical constituent of YLTZC was comprehensively characterized by UPLC-Q-TOF–MS, and a total of 66 constituents, including naringenin, and ferulic acid, were identified. The results of the study provide more information on the chemical substance basis for further studies of YLTZC. According to the C-T-P and PPI network analysis, naringenin and ferulic acid were discovered to be the core active constituents associated with the most targets, and the HLP-related core targets of YLTZC were ALB, TNF, IL6, and VEGFA. In this study, enrichment analysis of GO and KEGG pathways was performed on 52 common targets. GO function enrichment analysis results showed that YLTZC treatment of HLP may involve the following BP: lipid metabolic process, positive regulation of smooth muscle cell proliferation, positive regulation of MAPK cascade, and positive regulation of inflammatory response. We hypothesized that the response to lipid metabolic process and inflammatory may be the most important BP in the treatment of HLP by YLTZC. KEGG pathway enrichment analysis results demonstrated that HIF-1 signaling pathway, AGE-RAGE signaling pathway in diabetic complications, PPAR signaling pathway, PI3K-Akt signaling pathway, IR, and TNF signaling pathway were highly involved in YLTZC treatment of HLP. These pathways are highly associated with the regulation of angiogenesis and anti-inflammation systems, which may be significant pathways for the alleviation of the symptoms of HLP. Consequently, our findings suggest that the modulating the mechanism of angiogenesis and inhibiting inflammatory are potential therapeutic strategies of YLTZC for the treatment of HLP.

Naringenin has an antioxidative activity to relieve oxidative stress, alleviate IR, and inhibit the production of inflammatory mediators^35,36^. It has been shown that naringenin prevents HLP, IR, and atherosclerosis by decreasing TC, TG, and LDL and increasing HDL^37^. In addition, naringenin inhibited fibroblast activation and inflammatory cell recruitment. The mRNA and protein expression levels of TNF‑α, IL‑1β, IL‑6, and TGF‑β1 were downregulated following naringenin treatment^38^. Meanwhile, some studies have found that the effects of naringenin are related to the activation of PPARs, which can regulate hepatic lipid metabolism at the transcriptional level in human and rat by activating PPARα, PPARβ, or PPARγ, respectively, and reduces serum lipids in HLP rats^39,40^. Ferulic acid has been shown to have anti-HLP, antioxidant, and anti-inflammatory effects. Compared with the placebo, the ferulic acid supplementation showed a statistically significant decrease in TC, LDL-c, and TG, and increased HDL-c^41,42^. In addition, ferulic acid could inhibit the expression of several pro-inflammatory cytokines including IL-1β, TNF-α, IL-10, and IL-6, and also inhibit angiogenesis by reducing the expression of VEGFA mRNA^43–45^. In our study, we established a triton WR-1339-induced HLP mice model, supplied with core active constituents of YLTZC for confirming its hypolipidemic effect. The results showed that naringenin and ferulic acid treatment significantly decreased the levels of serum TC, TG, and LDL-c, and enhanced the level of serum HDL-c in HLP mice. Therefore, naringenin and ferulic acid as the two core active constituents may be the potential material basis for YLTZC to alleviate HLP.

On the one hand, YLTZC may treat HLP by regulating the mechanism of angiogenesis. VEGFA was found to be a significant factor in the regulation of vascular endothelial cells. Vascular remodeling during atherosclerosis was also associated with the expression of VEGFA, which has the effect of inducing angiogenesis, promoting their survival, and enhancing vascular permeability^46^.

On the other hand, YLTZC can alleviate HLP by regulating inflammatory and oxidative stress targets. For example, TNF-α is one of the most significant pro-inflammatory mediators and a critical factor in IR, which is involved in the pathophysiology of various CVDs^47^. IL6 is also a pro-inflammatory cytokine that plays a crucial role in inflammation, atherosclerosis, and thrombosis, and can influence the rate of lipid metabolism, specifically the metabolism of TCs and TGs^48^. ALB is an important substance for maintaining plasma colloid osmotic pressure, and studies have found that ALB levels are closely related to cardiac function, and play a critical role in regulating the osmotic pressure and metabolic processes^49^. Meanwhile, studies have also shown that ALB is the most important carrier/transporter protein in vivo and plays an essential role in plasma antioxidant activity. In addition, in the HLP model, the decreased levels of ALB may be closely associated with the decline of liver function leading to reduced liver production^50,51^. Therefore, we speculate that ALB is mainly used as a drug transport platform to treat HLP by YLTZC. PPARs play a vital role in regulating the systemic inflammatory response, and they also modulate several biological processes that perturbed obesity, including inflammation, lipid and glucose metabolisms^52^.

The HIF-1 signaling pathway is primarily involved in maintaining the steady state of oxygen in the body, and is also engaged in regulating angiogenesis and inflammation^47,53^. Activation of the AGE-RAGE signaling pathway can trigger the production of tissue factors, and inflammatory factors^54^. Moreover, the PPAR signaling pathway plays an essential part in cholesterol metabolism and cholesterol efflux^55^. IR is associated with hyperinsulinemia and HLP^56^. These pathways are highly involved in YLTZC treatment of HLP, among which the PI3K-Akt signaling pathway, PPAR signaling pathway, and IR play critical roles in insulin secretion and lipid metabolism. Furthermore, fluid shear stress and atherosclerosis, the HIF-1 signaling pathway, and the TNF signaling pathway are highly participated in angiogenesis, pro-inflammatory factor secretion, and vascular tone regulation^47,48^.

In summary, we identified 2 core active constituents in YLTZC and the potential targets and pathways underlying the effects of YLTZC in HLP using the network pharmacology method. The results identified some pathways and biological processes that could be related to the lipid-lowering effects of YLTZC. To further validate the feasibility of network pharmacology analysis, IL-6, TNF-α, VEGFA, and ALB were selected as candidate targets of YLTZC against HLP. The molecular docking verified that the core active constituents have good binding properties with the core targets. In vivo experiment, compared with the control group, the mRNA expression of IL-6, VEGFA, and TNF-α mRNA were significantly increased (*p* < 0.01), and decreased the mRNA expression of ALB (*p* < 0.01) in the model group. However, compared with the model group, the naringenin and ferulic acid groups inhibited the mRNA expression of IL-6, VEGFA, and TNF-α (*p* < 0.01), and promoted the mRNA expression of ALB (*p* < 0.05,* p* < 0.01). Therefore, we reasoned that YLTZC can effectively alleviate HLP by modulating the mechanism of angiogenesis and inhibiting inflammatory responses. The results of molecular docking and experimental verification were consistent with the predicted results of network pharmacology, indicating the accuracy of this method in screening the active constituents and action targets of YLTZC.

## Conclusion

In this study, we revealed the therapeutic effect and underlying mechanism of YLTZC against HLP based on network pharmacology, molecular docking, and experimental validation. 66 chemical constituents of YLTZC were rapidly characterized by UPLC-Q-TOF–MS/MS. Based on the network pharmacology approach, the core active constituents including naringenin and ferulic acid, as well as core targets such as ALB, TNF, IL6, and VEGFA were screened for YLTZC for the treatment of HLP. Functional enrichment analysis through GO and KEGG pathways demonstrated the regulation of lipid metabolism, angiogenesis, and anti-inflammation systems, which may be important pathways in alleviating HLP. Molecular docking verified the possibility of the core active constituents binding to the core targets. In vivo experiments further showed that the hypolipidemic mechanisms of YLTZC were associated with the down-regulation of TNF-α, IL6, and VEGFA mRNA expression levels, and the up-regulation of ALB mRNA expression levels. Collectively, this work demonstrated that YLTZC may act against HLP by modulating the mechanism of angiogenesis and inhibiting inflammatory responses, and provided an efficient way to understand the active constituents and underlying mechanisms of YLTZC.

## Supplementary Information

Supplementary Figure S1.

Supplementary Table S1.

## Data Availability

The datasets used and/or analysed during the current study were available from the corresponding author on reasonable request. The main supporting data can be found in the supplementary material of the article.
