# Supplementary material for: Deciphering the mechanisms of Yinlan Tiaozhi capsule in treating hyperlipidemia by combining network pharmacology, molecular docking and experimental verification
Source: Sci Rep. 2023 Apr 19;13:6424. doi: 10.1038/s41598-023-33673-3 (PMC10115829; doi:10.1038/s41598-023-33673-3)
Supplement: Supplementary file 1 — Supplementary Figure S1. [file 41598_2023_33673_MOESM1_ESM.pdf]

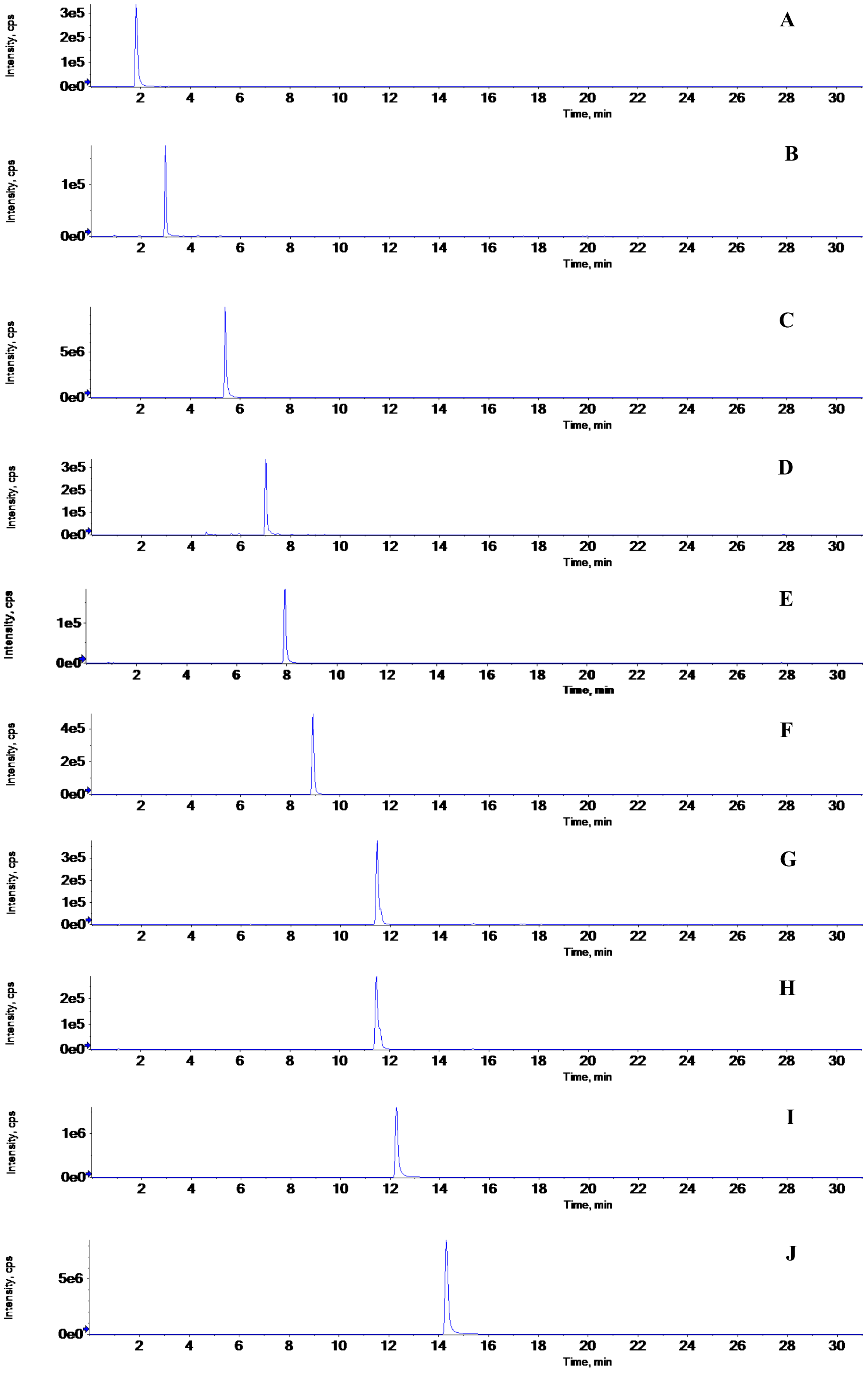
A
Intensity, cps
3e5
2e5
1e5
0e0
2 4 6 8 10 12 14 16 18 20 22 24 26 28 30
Time, min
B
Intensity, cps
1e5
0e0
Time, min
C
Intensity, cps
5e6
0e0
Time, min
D
Intensity, cps
3e5
2e5
1e5
0e0
Time, min
E
Intensity, cps
1e5
0e0
Time, min
F
Intensity, cps
4e5
2e5
0e0
Time, min
G
Intensity, cps
3e5
2e5
1e5
0e0
Time, min
H
Intensity, cps
2e5
1e5
0e0
Time, min
I
Intensity, cps
1e6
0e0
Time, min
J
Intensity, cps
5e6
0e0
Time, min

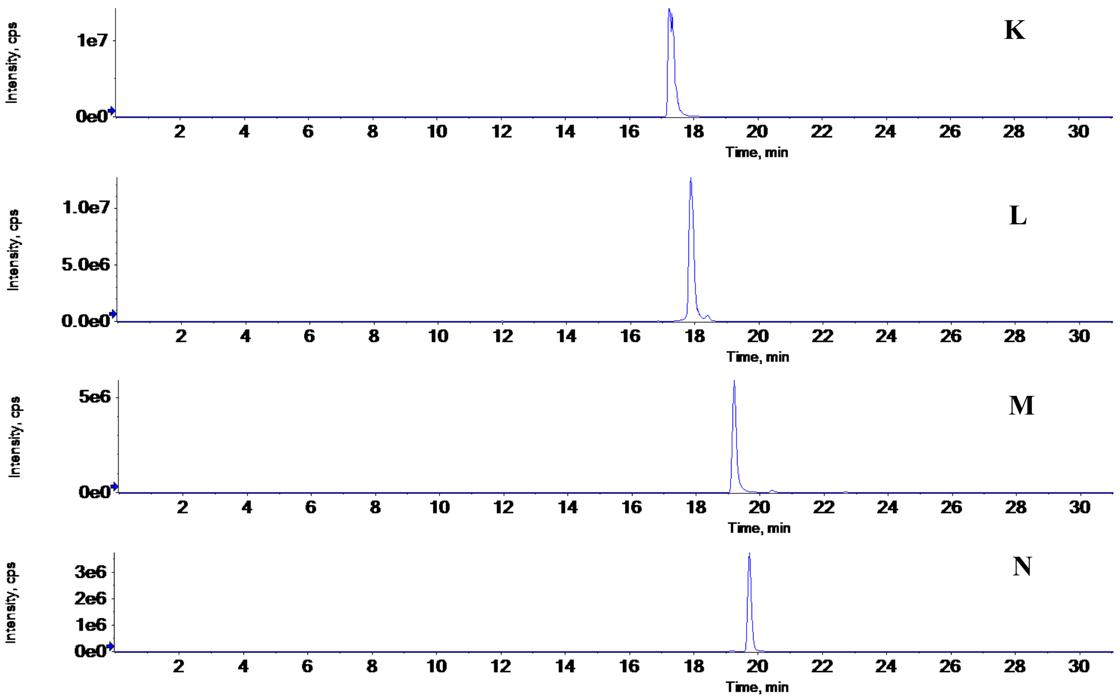

Fig. S1 The extracted ion chromatogram of reference

A. Gallic acid B. Protocatechuic acid C. Vicenin-2 D. Rutin E. Ferulic acid F. Naringin G. Ginkgolide A H. Ginkgolide B I. Luteolin J. Naringenin K. Isomeranzin L. Limonin M. Chrysin N. Pinocembrin
